# Caenorhabditis elegans HCF-1 Functions in Longevity Maintenance as a DAF-16 Regulator

**DOI:** 10.1371/journal.pbio.0060233

**Published:** 2008-09-30

**Authors:** Ji Li, Atsushi Ebata, Yuqing Dong, Gizem Rizki, Terri Iwata, Siu Sylvia Lee

**Affiliations:** Department of Molecular Biology and Genetics, Cornell University, Ithaca, New York, United States of America; The Salk Institute, United States of America

## Abstract

The transcription factor DAF-16/forkhead box O (FOXO) is a critical longevity determinant in diverse organisms, however the molecular basis of how its transcriptional activity is regulated remains largely unknown. We report that the Caenorhabditis elegans homolog of host cell factor 1 (HCF-1) represents a new longevity modulator and functions as a negative regulator of DAF-16. In C. elegans, *hcf-1* inactivation caused a *daf-16*-dependent lifespan extension of up to 40% and heightened resistance to specific stress stimuli. HCF-1 showed ubiquitous nuclear localization and physically associated with DAF-16. Furthermore, loss of *hcf-1* resulted in elevated DAF-16 recruitment to the promoters of its target genes and altered expression of a subset of DAF-16-regulated genes. We propose that HCF-1 modulates C. elegans longevity and stress response by forming a complex with DAF-16 and limiting a fraction of DAF-16 from accessing its target gene promoters, and thereby regulates DAF-16-mediated transcription of selective target genes. As HCF-1 is highly conserved, our findings have important implications for aging and FOXO regulation in mammals.

## Author Summary

One of the key molecules that modulate longevity in evolutionarily diverse organisms is the transcription factor DAF-16/FOXO. Despite its importance in aging and other biological processes, how DAF-16/FOXO activity is regulated in the nucleus is largely unknown. We report a new player important for aging modulation, the nematode homolog of host cell factor 1 (HCF-1), and show that it functions as a negative regulator of DAF-16. In worms, HCF-1 inactivation extends lifespan up to 40% and increases resistance to specific stress stimuli. To affect lifespan and stress response, HCF-1 requires the activity of DAF-16. We show that the HCF-1 protein is expressed in the nucleus and partners with DAF-16 in worms. Furthermore, we demonstrate that loss of HCF-1 results in elevated levels of DAF-16 at the promoters of its target genes and altered expression of a subset of DAF-16-regulated genes. We propose that HCF-1 modulates longevity and stress response by binding to DAF-16 and preventing the transcription factor from accessing its target gene promoters, thereby regulating the expression of DAF-16 target genes. As HCF-1 is highly conserved, our findings have important implications for aging and FOXO regulation in humans.

## Abstract 3

Caenorhabditis elegans HCF-1 is a new longevity factor that functions to antagonize the transcriptional activities of DAF-16/FOXO by forming a complex with DAF-16/FOXO and preventing a fraction of DAF-16/FOXO from accessing its target gene promoters.

## Introduction

Recent studies in various model system have revealed multiple evolutionarily conserved genes and genetic pathways important for longevity [1–6]. One of the best characterized longevity determinants is the forkhead box O (FOXO) family of transcription factors, which function as major effectors of the insulin/insulin-like growth factor (IGF)-1-like signaling (IIS) cascade. The IIS pathway is highly conserved and has been shown to modulate longevity in Caenorhabditis elegans, *Drosophila*, and mice [7]. Activation of the insulin/IGF receptor tyrosine kinases triggers a kinase cascade, involving the phosphoinositide 3-kinase (PI3K) and the serine/threonine kinases AKT-1 and AKT-2, which culminates in the cytoplasmic sequestration and inhibition of the FOXO transcription factors [7]. In addition to aging, IIS is also critical for regulating development, metabolism, and stress response. In C. elegans, reduced signaling of the IIS pathway, such as that caused by mutations in the IIS receptor *daf-2* or the PI3K *age-1,* results in a dramatic increase in lifespan, heightened resistance to a wide variety of environmental stress stimuli, and altered metabolism and development [8,9]. Loss of *daf-16*, the C. elegans homolog of FOXO, completely suppresses all the phenotypes associated with IIS deficiency in C. elegans [10–12], indicating *daf-16* to be the major effector of IIS in worms.

From C. elegans to mammals, DAF-16/FOXO is emerging as a master regulator that is capable of responding to diverse environmental stimuli and coordinating development, metabolism, and stress response [1,13]. In addition to the IIS pathway, DAF-16/FOXO also responds to many other signaling cascades. Recent findings reveal that different signals induce distinct modifications of DAF-16/FOXO, which can impact the expression level, subcellular localization, and/or transcriptional activities of DAF-16/FOXO, leading to expression changes of selective DAF-16/FOXO target genes and specific cellular responses [13,14]. Mammalian FOXOs have been shown to regulate the expression of antioxidant enzymes, gluconeogenic enzymes, cell cycle regulators, and apoptotic genes [13]. Similarly, C. elegans DAF-16 regulates the expression of a large number of target genes, including those involved in metabolism, stress response, and immunity [15–17]. DAF-16 is thought to promote survival and longevity by mounting a robust response to various stresses, infection, and toxic compounds. Therefore, the precise control of DAF-16 transcriptional activities is a key regulatory step for longevity determination.

Similar to other DNA binding transcription factors, one way for DAF-16/FOXO to achieve specificity in gene regulation depends on its functional interactions with transcriptional co-regulators. Recent findings have revealed several nuclear factors that cooperate with DAF-16/FOXO to regulate gene expression. In mammals, FOXOs can be deacetylated by the protein deacetylase SIRT1 [18–22]. SIRT1-mediated deacetylation leads to promotion of FOXO activation of stress response genes and concurrent inhibition of FOXO-regulated apoptotic genes [18]. Interestingly, SIRT1 homologs in yeast (*Sir2*), C. elegans (*sir-2.1*), and *Drosophila* (*dSir2*) are important longevity determinants as their over-expression results in longevity increase [23]. In C. elegans, SIR-2.1 forms a protein complex with DAF-16 [24,25] and requires DAF-16 activity to modulate lifespan [26]. Furthermore, the nuclear factor SMK-1 and the β-catenin homolog BAR-1 have recently been shown to promote DAF-16-mediated transcription [27,28]. C. elegans lacking *smk-1* or *bar-1* show shortened lifespan, similar to that of worms lacking *daf-16*. BAR-1 cooperates with DAF-16 to elicit proper oxidative stress response [28], and SMK-1 is important for the roles of DAF-16 in both lifespan modulation and specific stress response [27].

Whereas SIR-2.1, SMK-1, and BAR-1 represent putative positive regulators of DAF-16 in C. elegans, nuclear factors that negatively regulate DAF-16 are largely unknown. Since DNA binding transcription factors are often regulated by the interplay between positive and negative regulators, the characterization of DAF-16 negative regulators will be essential for the further elucidation of DAF-16 regulation. In this paper, we report that the C. elegans homolog of host cell factor 1 (HCF-1) represents a new longevity determinant and functions as a negative regulator of DAF-16. HCF-1 belongs to a family of highly conserved proteins [29]. Loss of *hcf-1* in C. elegans induces substantial lifespan extension of up to 40% and robust resistance to specific stress stimuli. For *hcf-1* to modulate lifespan and stress response, it requires the activity of *daf-16*. In delineating the mechanism by which HCF-1 regulates DAF-16, we found HCF-1 to be a ubiquitously expressed nuclear protein that physically associates with DAF-16. Moreover, loss of *hcf-1* led to increased recruitment of some DAF-16 to its target gene promoters and altered expression of a subset of DAF-16-regulated genes. Given the genetic and biochemical data, we propose that HCF-1 modulates lifespan and stress responses by forming a complex with DAF-16 and restricting the recruitment of a fraction of DAF-16 to its target gene promoters, thereby regulating DAF-16-mediated transcription of specific target genes. Considering that HCF-1 and DAF-16 are both highly conserved through evolution, our findings suggest that HCF-1 likely also regulates FOXO activities and is important for aging in diverse organisms.

## Results

### *hcf-1* Is a Novel Longevity Gene

**Figure 1:**
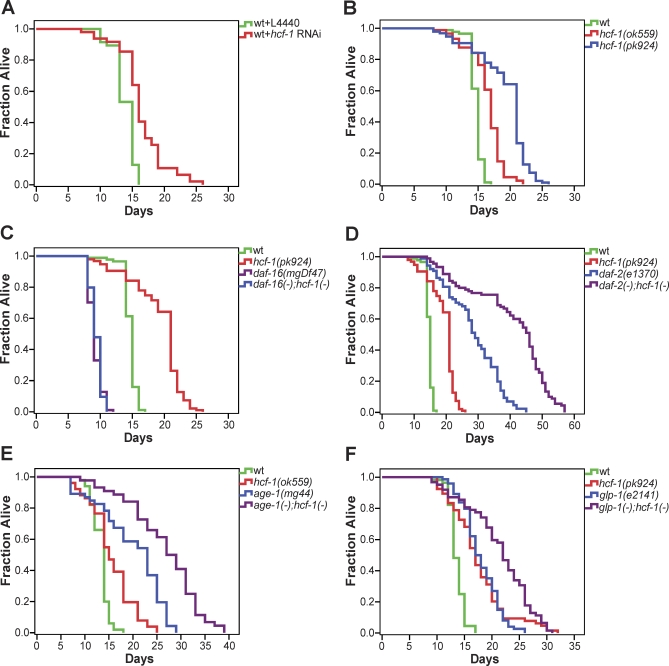
*hcf-1* Modulates Lifespan by Acting Upstream of *daf-16*, but in Parallel to the IIS and Germline Signaling Pathways Lifespan of (A) wild-type worms treated with *hcf-1* RNAi, (B) the *hcf-1(ok559)* and *hcf-1(pk924)* deletion mutants, (C) the *daf-16(mgDf47);hcf-1(pk924)* double mutant, (D) the *daf-2(e1370);hcf-1(pk924)* double mutant, (E) the *age-1(mg44);hcf-1(ok559)* double mutant, (F) the *glp-1(e2141);hcf-1(pk924)* double mutant worms. Each of the lifespan experiments was repeated at least two independent times with similar results. Data from representative experiments are shown. Quantitative data and statistical analyses for the experiments shown here are included in Table 1.

In a recent genome-wide RNA interference (RNAi) screen for new longevity genes [30], we found that RNAi knock down of the *hcf-1* gene consistently caused C. elegans to live ∼20%–30% longer than control RNAi treated worms (Figure 1A; Table 1). *C. elegans hcf-1* encodes a protein that is highly conserved through evolution, but its biological function is just beginning to be elucidated. Mammalian HCF-1 was first identified to be the host cell factor essential for stabilizing the transcriptional complex involving the herpes simplex virus (HSV) VP-16 transcription factor [29]. Mammalian HCF-1 has subsequently been shown to play key roles in cell cycle progression, both at the G1/S transition, and at M phase and cytokinesis [31–33]. In eliciting its diverse biological roles, mammalian HCF-1 acts by binding to and regulating many different transcription and chromatin factors and assembling appropriate protein complexes for context-dependent gene expression regulation [29]. C. elegans HCF-1 has been shown to complement some of the transcriptional role of mammalian HCF-1 [34] and to also be required for proper cell cycle progression in worms (Figure S2) [35]. Our data ascribe a new longevity function for HCF-1. To further investigate a role of *hcf-1* in C. elegans longevity, we obtained two mutant alleles (*ok559* and *pk924*) [35] of *hcf-1* and examined their lifespan. The *hcf-1(ok559)* mutant has an in-frame deletion that should truncate the N-terminal half of the protein, and the *hcf-1(pk924)* mutant has a large deletion that should result in a frame shift leading to an early stop codon, and likely represents a null mutant (Figure S1) [35]. As expected on the basis of our observation with the *hcf-1* RNAi worms, we detected up to 40% lifespan extension in the *hcf-1* mutants compared to wild-type worms (Figure 1B; Table 1). In general, the *hcf-1(pk924)* mutant lives slightly longer than the *hcf-1(ok559)* mutant (Table 1), consistent with the possibility that the *pk924* allele is a more severe mutation. We confirmed that the prolonged lifespan associated with the *hcf-1* mutants is due to *hcf-1* deficiency by demonstrating that expression of a C-terminal green fluorescent protein (gfp)-tagged *hcf-1* transgene (*hcf-1::gfp*) was able to partially restore normal lifespan in the *hcf-1(pk924)* mutant (Table S2).

**Table 1:**
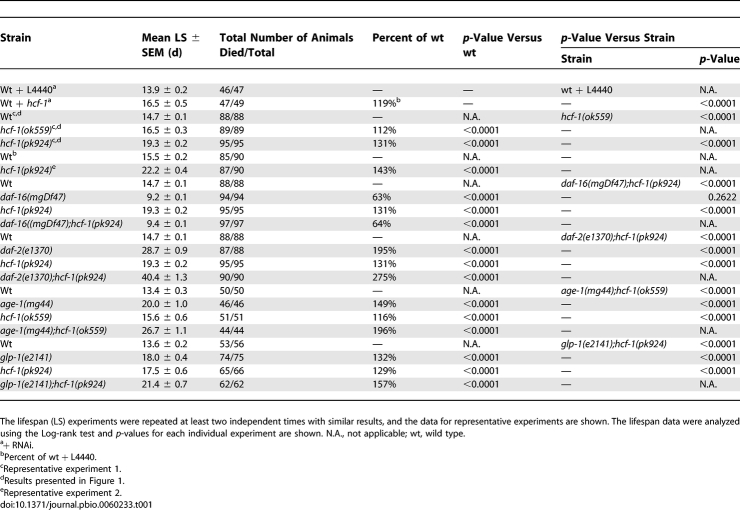
Inactivation of *hcf-1* Results in Lifespan Increase That Is Completely Dependent on *daf-16*, but Likely Independent of the IIS and Germline Pathways

### HCF-1 Is Ubiquitously Expressed and Localizes to the Nucleus

**Figure 2:**
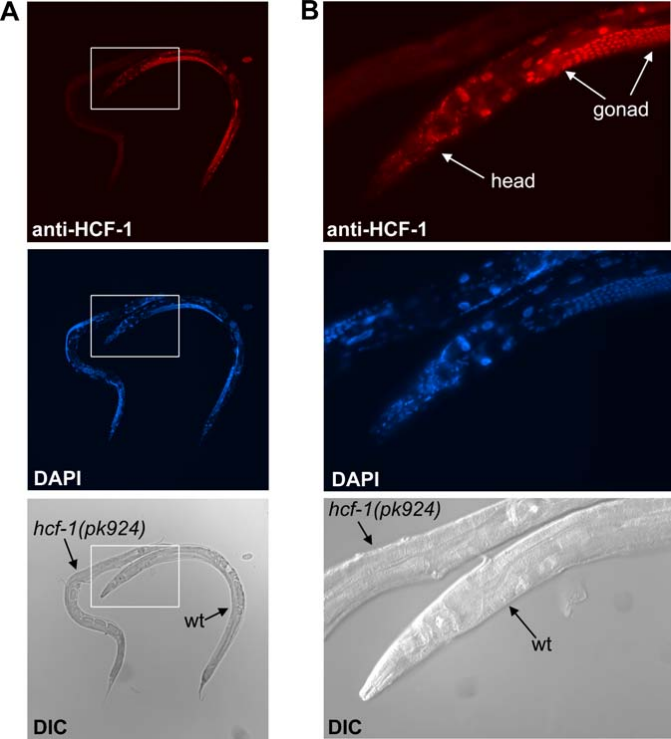
HCF-1 Is a Ubiquitously Expressed Nuclear Protein (A) Gravid adults of wild-type and *hcf-1(pk924);sur-5::gfp* mutant worms were immunostained using an affinity-purified HCF-1 antibody. To ensure identical staining conditions, worms from both strains were processed on the same slide. The *hcf-1(pk924)* mutants were marked by SUR-5::GFP to distinguish them from wild-type worms. Endogenous HCF-1 was found to localize in the nucleus of most, if not all, somatic and germline cells in wild-type worms. The *hcf-1(pk924)* mutant worms marked by SUR-5::GFP showed only background signal. DAPI staining was used to indicate the nucleus. Photos were taken at 200× magnification. (B) A magnified image of the head region of the wild-type worm shown in (A).

To further characterize HCF-1 in C. elegans, we examined the expression pattern of HCF-1 in worms. Using an affinity-purified polyclonal HCF-1 antibody in immunostaining assays, we observed prominent HCF-1 staining in the nuclei of most, if not all, somatic and germline cells in wild-type worms (Figure 2). The HCF-1 expression pattern detected in wild-type worms is highly specific because the *hcf-1(pk924)* and *hcf-1(ok559)* mutants only showed background fluorescence when they were examined using identical immunostaining conditions (Figure 2) (unpublished data). The nuclear localization of HCF-1 was consistently observed from embryo through larval and adult stages (Figure 2) (unpublished data) [35]. Moreover, we observed similar ubiquitous nuclear expression in worms expressing a low-copy number of a functional *hcf-1::gfp* transgene (Figure S3). Our results indicate that HCF-1 is ubiquitously expressed in the nucleus of C. elegans under normal culture condition. Mammalian HCF-1 is also predominantly a nuclear protein [36] and its nuclear localization is thought to be important for its role in gene expression regulation.

### *hcf-1* Modulates Lifespan in a *daf-16*-Dependent Manner

To characterize how *hcf-1* modulates C. elegans lifespan, we asked whether *hcf-1* may genetically interact with any known longevity factors in C. elegans. Because DAF-16/FOXO is one of the best characterized longevity determinants, we tested the epistatic relationship between *hcf-1* and *daf-16*. We created double mutants containing the *hcf-1(pk924)* or *hcf-1(ok559)* and the null *daf-16(mgDf47)* mutations. We found that the *daf-16(mgDf47);hcf-1(pk924)* or *daf-16(mgDf47);hcf-1(ok559)* double mutant had a lifespan indistinguishable from that of the *daf-16(mgDf47)* single mutant, which is ∼20% shorter than wild-type worms [37] (Figure 1C; Tables 1 and S1). We obtained similar results with *hcf-1(ok559)* mutant worms treated with *daf-16* RNAi (Table S1). Our results indicate that *hcf-1* requires the activity of *daf-16* to modulate longevity in C. elegans and suggest that *hcf-1* may be a novel upstream regulator of *daf-16*.

### *hcf-1* Likely Acts in Parallel to the IIS and Germline Pathways to Modulate Lifespan

Given the epistatic relationship between *hcf-1* and *daf-16*, we wondered whether *hcf-1* modulates C. elegans lifespan by functioning in the IIS pathway, a well-established upstream regulator of *daf-16*. To test this, we examined the genetic interactions between *hcf-1* and two major components of the IIS pathway: *daf-2*/insulin/IGF receptor and *age-1*/PI3K. We reasoned that if *hcf-1* normally affects C. elegans lifespan by acting in the IIS pathway, then loss of *hcf-1* would not have a major impact on the longevity of worms already lacking IIS signaling. We created double mutants containing the *hcf-1(pk924)* or *hcf-1(ok559)* and either the *daf-2(e1370)* or the *age-1(mg44)* mutations. Consistent with previous findings [11], the *daf-2(e1370)* temperature-sensitive mutant showed an ∼2-fold increase in lifespan compared to wild-type worms at the nonpermissive temperature 25 °C (Figure 1D; Tables 1 and S1). Interestingly, the *daf-2(e1370);hcf-1(pk924)* or *daf-2(e1370);hcf-1(ok559)* double mutant lived considerably longer than either the *daf-2(e1370)* or *hcf-1(pk924)* or *hcf-1(ok559)* single mutant (Figure 1D; Tables 1 and S1), and exhibited a lifespan increase that is greater than the sum of the effect for the two single mutations.

We obtained similar results with the *age-1(mg44);hcf-1(ok559)* double mutant. *age-1(mg44)* is a null mutant and, when maintained as a homozygous strain, exhibits an unconditional dauer arrest phenotype [10,38]. To avoid this, we collected *age-1(mg44)* or *age-1(mg44);hcf-1(ok559)* homozygous mutant adults born from *age-1(mg44)*/+ or *age-1(mg44)*/+;*hcf-1(ok559)* parents for lifespan analysis. These *age-1(mg44)* and *hcf-1(ok559)*;*age-1(mg44)* worms completely lacked zygotic *age-1* expression; however, they inherited sufficient maternal *age-1* message to develop normally [38]. Similar to previous results [38], the *age-1(mg44)* zygotic null mutant worms lived much longer than wild-type worms (Figure 1E; Table 1). The *age-1(mg44);hcf-1(ok559)* double mutant worms lived considerably longer than either the *age-1(mg44)* or *hcf-1(ok559)* single mutant (Figure 1E; Table 1), and exhibited a lifespan increase that is even greater than the sum of the effect for the single mutations. RNAi knock down of *daf-2* or *age-1* in the *hcf-1(ok559)* mutant gave similar results (Table S1). The genetic results described here indicate that loss of *hcf-1* and loss of IIS act synergistically to extend lifespan in C. elegans and suggest that *hcf-1* likely functions in a pathway in parallel to IIS. Although the *hcf-1(pk924)* mutation is a putative null mutation, the *daf-2(e1370)* mutation is a temperature sensitive mutation, and a small amount of maternal AGE-1 protein may have persisted in the *age-1(mg44);hcf-1(ok559)* double mutant, it remains possible that loss of *hcf-1* increases lifespan by further decreasing *daf-2* signaling.

Since germline proliferation has been implicated in C. elegans lifespan modulation, we wondered whether the extended lifespan of the *hcf-1* mutant is related to the brood size defect of this mutant (Figure S2) [35]. In this regard, we tested the lifespan of the double mutant *glp-1(e2141);hcf-1(pk924)*. The *glp-1(e2141)* mutant completely lacks germline cells at the nonpermissive temperature 25 °C and is long-lived [39]. If the *hcf-1* mutant is long-lived due to its partial defect in germline proliferation, then we would expect *hcf-1* deficiency not to affect the lifespan of worms completely lacking germline cells. We found that the *glp-1(e2141);hcf-1(pk924)* double mutant lived much longer than the *glp-1(e2141)* or *hcf-1(pk924)* single mutants at 25 °C (Figure 1F; Table 1). Therefore, loss of *hcf-1* can continue to extend the lifespan of worms that completely lack germline cells and are sterile, suggesting that *hcf-1* has a function in lifespan modulation that is beyond its role in germline and brood size regulation.

### Loss of *hcf-1* Promotes Resistance to Specific Environmental Stress

**Figure 3:**
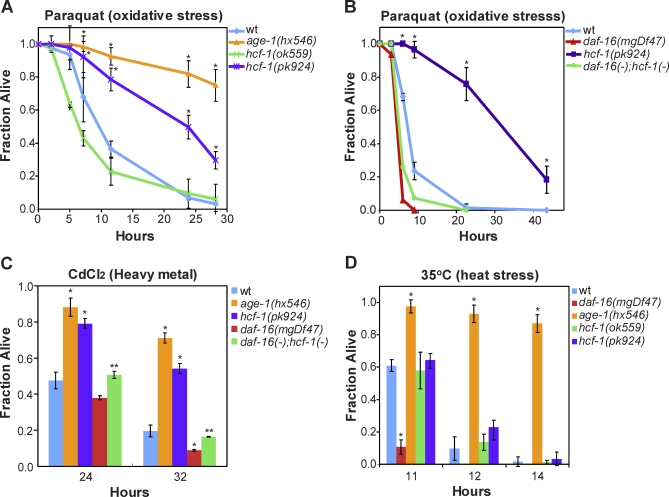
Loss of *hcf-1* Results in Heightened Resistance to Specific Environmental Stresses (A) The *hcf-1(pk924)* mutant worms exhibited increased survival in 200-mM paraquat compared to wild-type worms. (B) The enhanced paraquat resistance of *hcf-1(pk924)* was dependent on *daf-16*. (C) The *hcf-1(pk924)* mutant worms showed increased survival in CdCl_2_ (18mM) that was *daf-16* dependent. (D) The *hcf-1(pk924)* and *hcf-1(ok559)* mutants and wild-type worms showed similar survival kinetics when cultured at 35 °C. The *age-1(hx546)* and *daf-16(mgDf47)* worms were included as controls as they have been previously reported to be either resistant or sensitive to paraquat, heavy metal, or heat shock, respectively [41–43]. For the stress assays, duplicate to quadruplicate samples were examined for each strain. Mean fraction alive indicates the average survival among the multiplicates and error bars represent the standard deviation of the multiplicates. *p*-Value was calculated using Student's *t*-test. *, *p* < 0.05 when compared to wild-type (wt). **, *p* < 0.05 when compared to *hcf-1(pk924)*. Each of the stress assays was repeated at least two independent times with similar results, and the data of representative experiments are shown.

Since DAF-16/FOXO is well known to regulate stress responses [5,40,41], we tested whether the *hcf-1* mutants may exhibit differential response to environmental stress stimuli. To assay for a response to acute oxidative stress, we challenged wild-type or *hcf-1* mutant adult worms with a high-dose of paraquat, a superoxide-inducing agent, and monitored their survival. We found that the *hcf-1(pk924)* mutant worms were considerably more resistant to the paraquat treatment compared to wild-type worms at multiple time points throughout the experiment (Figure 3A). Moreover, the paraquat resistance of the *hcf-1(pk924)* mutants was dependent on *daf-16*, as the *daf-16(mgDf47);hcf-1(pk924)* double mutant was sensitive to paraquat, similar to that of the *daf-16(mgDf47)* single mutant (Figure 3B). Interestingly, the *hcf-1(ok559)* mutant worms had survival kinetics very similar to that of wild-type worms in the paraquat assay, suggesting that the *hcf-1(ok559)* mutant, while long-lived, was not more resistant to a high dose of paraquat treatment compared to wild-type worms. No HCF-1 protein is detected in the *hcf-1(pk924)* mutant, whereas some truncated protein accumulates in the *hcf-1(ok559)* mutant (Figure S1). Therefore, it is possible that resistance to a high level of oxidative stress only becomes apparent when HCF-1 is completely lost.

To assay for a response to heavy metal stress, we challenged wild-type or *hcf-1* mutant adult worms with cadmium [42] and monitored their survival. Similar to that observed with the paraquat assay, the *hcf-1(pk924)* mutant worms were more resistant to the cadmium exposure compared to wild-type worms at multiple time points (Figure 3C). The cadmium resistance of the *hcf-1(pk924)* mutant was also *daf-16*-dependent (Figure 3C).

To assay for a response to heat stress, we shifted adult wild-type and *hcf-1* mutant worms to 35 °C and monitored their survival. We found that the *hcf-1* mutants and wild-type worms behaved very similarly throughout the time course of the heat shock treatment (Figure 3D). As previously reported [43], the *age-1(hx546)* mutant worms survived much longer and the *daf-16(mgDf47)* mutant worms died much faster than wild-type worms at 35 °C. We therefore concluded that loss of *hcf-1* did not result in altered response to acute heat shock. Taken together, our results indicate that loss of *hcf-1* results in worms that are resistant to paraquat and cadmium exposure, but not to heat shock, suggesting that *hcf-1* is required for specific stress response.

Considering that DAF-16 also plays a key role in dauer formation and fat metabolism in C. elegans [10,44,45], we tested whether the *hcf-1* mutants exhibit any dauer or fat phenotypes. We found that the *hcf-1(ok559)* mutant exhibits no dauer phenotype, whereas the *hcf-1(pk924)* null mutant exhibits a weak dauer exit phenotype. When monitored at 25 °C, a typical temperature for testing a strong dauer phenotype, both *hcf-1* mutants developed normally, whereas the *daf-2(e1370)* mutant formed 100% dauer (Table 2). When monitored at 27 °C, a temperature commonly used to test for a weak dauer formation phenotype, neither *hcf-1* mutant behaved differently from wild-type worms (Table 2). Lastly, we tested the *daf-2;hcf-1* double mutants at 22 °C, which represents a well-established sensitized condition [46] for assaying a weak dauer recovery phenotype. Single and double mutant worms harboring the *daf-2(e1370)* mutation were incubated at the semi-nonpermissive temperature of 22 °C. Under this condition, the *daf-2(ts)* worms enter dauer for ∼3 d and then recover to become reproductive adults. We found that the *hcf-1(pk924)* mutation prevented dauer exit, whereas the *hcf-1(ok559)* mutation had no effect (Table 2). These results indicate that loss of *hcf-1* is associated with a weak dauer phenotype. Using the vital dye Nile Red, which stains lipid droplets in worms and represents a sensitive way to monitor fat storage [47], we did not detect any substantial differences in fat storage between *hcf-1* mutants and wild-type worms (Figure S4).

**Table 2:**
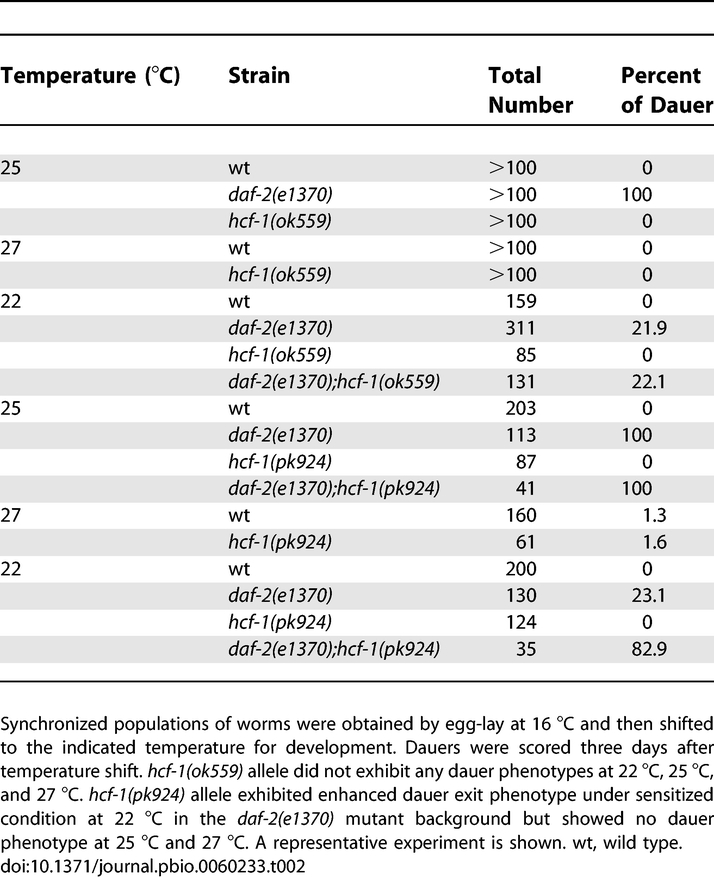
Inactivation of *hcf-1* Results in a Weak Dauer Exit Phenotype.

### HCF-1 Regulates the Expression of a Subset of DAF-16 Regulated Genes

**Figure 4:**
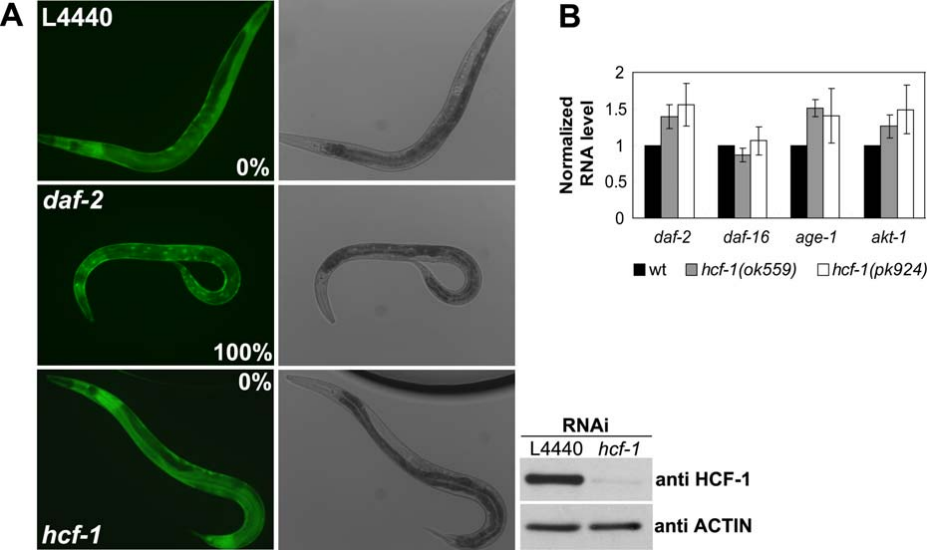
Loss of *hcf-1* Does Not Result in Altered DAF-16 Subcellular Localization or a Change in DAF-16 Expression Level (A) Transgenic worms over-expressing DAF-16::GFP (*daf-16(mgDf47);xrIs87*) were treated with empty vector L4440 control RNAi, *hcf-1* RNAi, or *daf-2* RNAi at 16 °C for 5 d. DAF-16::GFP exhibited diffuse expression pattern in both the control RNAi and the *hcf-1* RNAi knock down worms. *hcf-1* RNAi was able to substantially reduce HCF-1 levels (bottom right panel). *daf-2* RNAi was included as a positive control as it is known to stimulate robust nuclear localization of DAF-16::GFP. Photos showed the DAF-16::GFP expression pattern and DIC images of live day 2 gravid adults. Nuclear localization was verified using DIC. A total of ∼60–70 worms were scored, and the percentage of worms showing DAF-16::GFP nuclear localization was shown in the photo. Worm extracts made from the DAF-16::GFP worms treated with control or *hcf-1* RNAi were immunoblotted using anti-HCF-1 antibody ([A], bottom right panels). (B) The RNA levels of *daf-16*, *daf-2*, *age-1*, and *akt-1* in wild-type, *hcf-1(ok559)*, and *hcf-1(pk924)* worms were quantified using qRT-PCR. The data for three independent experiments were pooled, and the mean normalized RNA level and standard error of the mean (SEM) for each gene in the *hcf-1* mutant and wild-type worms are shown. *act-1* was used as an internal control, and the RNA level of each gene was normalized to the *act-1* level. The mean normalized RNA level for each gene in wild-type (wt) worms was set as 1. None of the genes tested showed any significant expression change in the *hcf-1* mutants compared to wild-type worms.

Our genetic data suggest that *hcf-1* acts upstream of *daf-16* to affect C. elegans lifespan and stress responses. Since increased DAF-16 nuclear localization and transcriptional activities have been shown to extend lifespan in C. elegans, we tested whether HCF-1 may regulate the localization, expression level, and/or transcriptional activities of DAF-16. Using transgenic worms expressing GFP-fused DAF-16 to monitor the subcellular localization of DAF-16, we did not detect any altered DAF-16 localization in *hcf-1*-deficient worms (Figure 4A). Using quantitative reverse transcription PCR (qRT-PCR) and immunoblotting to examine the RNA and protein expression levels of *daf-16* and the major components of the IIS pathway, including *daf-2*, *age-1*, and *akt-1*, we observed no obvious differences in their expression levels in the *hcf-1(ok559) and hcf-1(pk924)* mutants compared to wild-type worms (Figure 4B) (unpublished data). Taken together, our results suggest that HCF-1 is not likely to affect the subcellular localization or the expression level of DAF-16.

**Figure 5:**
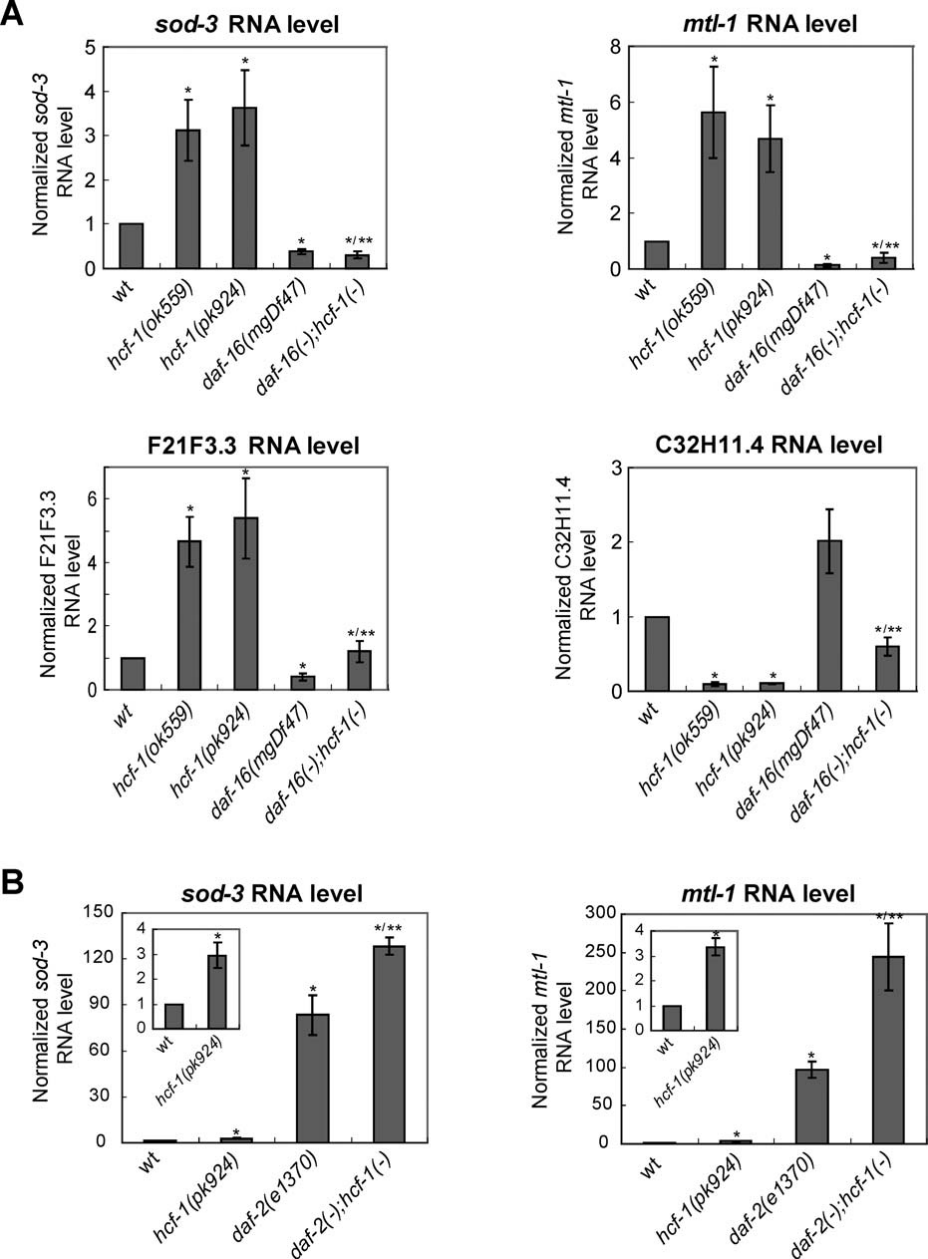
Loss of *hcf-1* Promotes the DAF-16 Transcriptional Regulation of Several Target Genes (A) The expression of *sod-3*, *mtl-1*, and *F21F3.3* was elevated and that of *C32H11.4* was repressed in the *hcf-1* mutants. The elevated expression of *sod-3* and *mtl-1* in the *hcf-1* mutants was completely dependent on *daf-16*; that of *F21F3.3* and *C32H11.4* was partially dependent on *daf-16*. *, *p* < 0.05 when compared to wild-type (wt). **, *p* < 0.05 when compared to *hcf-1(ok559)*. (B) The expression of *sod-3* and *mtl-1* in the *daf-2(e1370);hcf-1(pk924)* double mutant showed synergistic up-regulation when compared to the expression in either *hcf-1(pk924)* or *daf-2(e1370)* single mutant. *, *p* < 0.05 when compared to wild-type (wt). **, *p* < 0.05 when compared to *daf-2(e1370)*. The quantitative data are summarized in Tables 3–5. The RNA levels of *sod-3*, *mtl-1*, *F21F3.3*, and *C32H11.4* were quantified using qRT-PCR and normalized to the internal control *act-1*. The data for at least three independent experiments were pooled, and the mean normalized RNA level and SEM for each gene in the indicated strains are shown. The mean normalized RNA level for each gene in wt worms was set as 1. *p*-Value was calculated using Student's *t*-test. For *sod-3* or *mtl-1* expression, we analyzed for a synergistic effect in *daf-2(e1370);hcf-1(pk924)* compared to *daf-2(e1370)* or *hcf-1(pk924)* using two-way ANOVA analysis.

To test whether HCF-1 may affect the transcriptional activities of DAF-16, we measured the message levels of DAF-16-regulated genes in the *hcf-1* mutant and wild-type worms using qRT-PCR. *sod-3*, which encodes an iron/manganese superoxide dismutase, is one of the best characterized DAF-16 target genes [17,40] and its transcription is directly up-regulated by DAF-16 [48]. Interestingly, we found that the RNA level of endogenous *sod-3* was significantly elevated 3–4-fold in both the *hcf-1(ok559)* and *hcf-1(pk924)* mutants as compared to wild-type worms (Figure 5A). The elevated expression of *sod-3* in the *hcf-1* mutant worms is completely dependent on *daf-16* because in the *daf-16(mgDf47);hcf-1(ok559)* double mutant, the level of *sod-3* expression remained low and was similar to that seen in the *daf-16(mgDf47)* single mutant worms (Figure 5A). In corroboration of our qRT-PCR results, we observed elevated levels of GFP expression in *Psod-3::gfp* transgenic worms, which express a GFP reporter driven by the *sod-3* promoter, in *hcf-1(pk924)* mutant background (Figure S5).

**Table 3:**
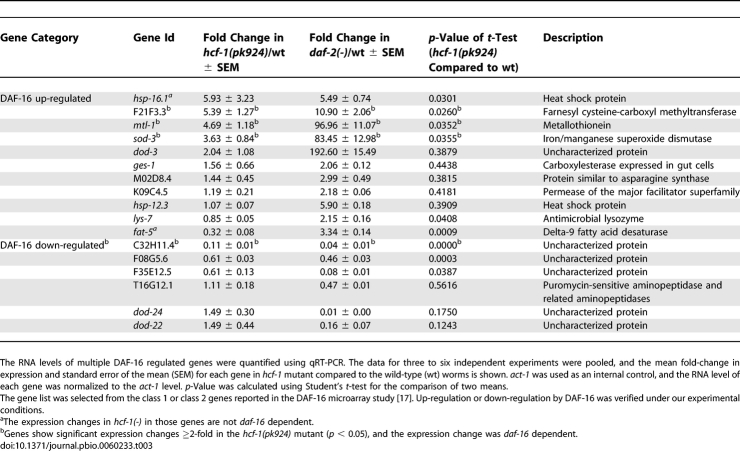
Inactivation of *hcf-1* Results in Significant Expression Changes of a Subset of DAF-16 Regulated Genes

To investigate whether *hcf-1* may generally affect the transcriptional activities of DAF-16, we surveyed additional DAF-16-regulated genes as reported in previous microarray studies [17]. The previous studies have focused on DAF-16 targets that are responsive to IIS and we verified that all the genes we chose to test exhibit *daf-2/daf-16* responsiveness under our assaying conditions (Table 3). Among the 11 DAF-16-activated genes examined, we found that in addition to *sod-3*, the expression levels of *mtl-1*, which encodes a metallothionein, and *F21F3.3*, which encodes an farnesyl cysteine-carboxyl methyltransferase, showed a statistically significant, greater than 2-fold up-regulation in the *hcf-1* mutants compared to wild-type worms (Figure 5A; Table 3). The elevated expression of *mtl-1* in *hcf-1* mutant worms is completely dependent on *daf-16* (Figure 5A; Table 4) and that of *F21F3.3* is partially dependent on *daf-16* (Figure 5A; Table 4). Among the six DAF-16-repressed genes examined, we found that the expression level of *C32H11.4*, which encodes a protein of unknown function, showed a statistically significant, greater than 2-fold down-regulation in the *hcf-1* mutants compared to wild-type worms (Figure 5A; Table 3). Similar to that of *F21F3.3*, the repressed expression of *C32H11.4* is partially dependent on *daf-16* (Figure 5A; Table 4). The partial dependency suggests that additional factors might cooperate with DAF-16 to regulate *F21F3.3* and *C32H11.4* expression in the *hcf-1* mutants.

**Table 4:**
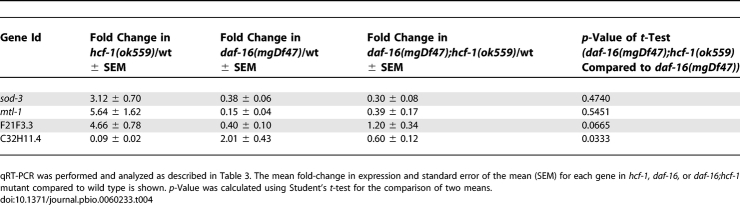
Expression Changes of Several HCF-1 Regulated Genes Are *daf-16* Dependent

We also noticed that the expression of *hsp-16.1*, which encodes a heat shock protein, and *fat-5*, which encodes a delta-9 fatty acid desaturase, was significantly up-regulated and repressed, respectively, in the *hcf-1* mutants (Table 3). However, the altered expression of *fat-5* and *hsp-16.1* in the *hcf-1* mutants did not require *daf-16* (unpublished data). These results suggest that although *hsp-16.1* and *fat-5* are robust DAF-16 targets in response to *daf-2* signaling (Table 3), they do not appear to be significantly regulated by DAF-16 in response to *hcf-1* deficiency. Moreover, whereas *fat-5* is activated by DAF-16 in response to reduced *daf-2* signaling, its expression was repressed in the *hcf-1* mutant in a *daf-16*-independent manner. Many of the DAF-16 downstream genes we surveyed are likely regulated by multiple different transcription factors. For example, *hsp-16.1* is likely also regulated by the heat shock factor HSF-1. It is possible that in the *hcf-1* mutants, different transcription factor(s) play a major role in mediating the expression change of *hsp-16.1* and *fat-5*, and DAF-16 only plays a minor role or is not involved in their gene expression regulation. The expression of the remaining DAF-16 target genes either did not show a significant change in the *hcf-1* mutants, or their expression change was less than 2-fold.

Consistent with our genetic data in which *hcf-1* appears to act in parallel to *daf-2* signaling to affect lifespan, the expression of *sod-3* and *mtl-1* was synergistically up-regulated in the *daf-2(e1370);hcf-1(pk924)* double mutant compared to either *daf-2(e1370)* or *hcf-1(pk924)* single mutant (Figure 5B; Table 5). Taken together, our results suggest that *hcf-1* is only able to affect the expression of selective DAF-16 target genes. Moreover, our data indicate that *hcf-1* inactivation leads to *daf-16*-dependent up-regulation of three different DAF-16 activated genes and *daf-16*-dependent down-regulation of a DAF-16 repressed gene, suggesting that *hcf-1* normally participates in the inhibition of DAF-16 transcriptional activity. Importantly, the dependence of *hcf-1* on *daf-16* to elicit gene expression changes correlates with the requirement of *daf-16* in *hcf-1*-mediated lifespan modulation, suggesting that the ability of HCF-1 to regulate DAF-16 transcriptional activity is likely linked to its role in longevity. Considering that C. elegans HCF-1 is normally localized to the nucleus, and that its mammalian homolog has a known role in gene expression regulation, we hypothesize that HCF-1 modulates C. elegans lifespan by regulating the transcriptional activity of DAF-16 at a subset of target genes that are particularly important for stress response and longevity assurance.

**Table 5:**
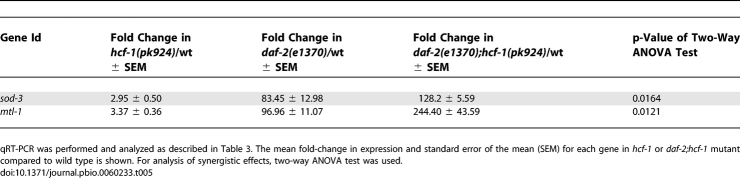
Inactivation of *hcf-1* and *daf-2* Results in Synergistic Regulation of Some DAF-16 Target Genes

### HCF-1 Forms a Protein Complex with DAF-16

**Figure 6:**
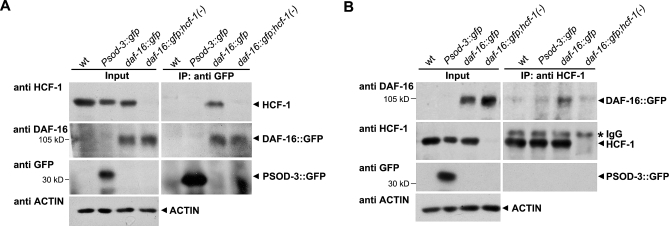
HCF-1 Forms a Protein Complex with DAF-16 in C. elegans Worm extracts were made from mixed stage worms and subjected to immunoprecipitation. Extracts from wild-type (wt), *Psod-3::gfp*, *daf-16::gfp (daf-16(mu86);muIs71)*, and *daf-16::gfp;hcf-1(-) (daf-16(mu86);hcf-1(ok559);muIs71)* worms were either immunoprecipitated using anti-GFP antibody (A) or anti-HCF-1 antibody (B). The immunoprecipitated protein complexes were subsequently immunoblotted using anti-HCF-1, anti-DAF-16, or anti-GFP antibodies. *Psod-3::gfp* worms were used as a negative control to indicate that there was no interaction between the GFP tag and HCF-1. For input, 50 μg of total protein was loaded per lane. For immunoprecipitation, 2 mg of total protein was used.

We next examined how HCF-1 might affect the transcriptional activity of DAF-16. In mammalian cells, HCF-1 is thought to regulate gene expression by binding to various transcription and chromatin factors [33,49–51]. In C. elegans, DAF-16 localizes to both cytoplasm and nucleus under normal culture condition, and HCF-1 appears predominantly nuclear in most cells (Figure 2) [35,37,52], suggesting that DAF-16 and HCF-1 co-localize in the nucleus (Figure S6). We therefore tested whether C. elegans HCF-1 may physically associate with DAF-16. Since we did not have an antibody that could detect endogenous DAF-16 robustly, we performed co-immunoprecipitation (co-IP) experiments using worm strains that lack endogenous DAF-16, but carry in low-copy number a functional gfp-fused *daf-16* transgene (*daf-16(mu86);muIs71*) [37]. We found that when an affinity-purified HCF-1 antibody was used to immunoprecipitate HCF-1 from extracts of the *daf-16::gfp* transgenic worms, DAF-16::GFP was co-immunoprecipitated (Figure 6B). This interaction appeared specific because when co-IP experiments were done using extracts of *daf-16::gfp* worms that also harbor the *hcf-1(ok559)* deletion (*daf-16(mu86);hcf-1(ok559);muIs71*), we were not able to immunoprecipitate HCF-1 or to co-immunoprecipitate DAF-16::GFP (Figure 6B). Furthermore, using identical co-IP conditions, we did not recover the irrelevant Psod-3::GFP (a GFP reporter driven by the *sod-3* promoter) upon HCF-1 immunoprecipitation, indicating that the protein–protein interaction detected between DAF-16::GFP and HCF-1 is not likely mediated by the GFP tag. We obtained similar results using reciprocal co-IP experiments (Figure 6A). Our results suggest that HCF-1 is able to form a specific protein complex with DAF-16 in C. elegans.

### Loss of *hcf-1* Leads to Enhanced Enrichment of DAF-16 on Its Target Gene Promoters

**Figure 7:**
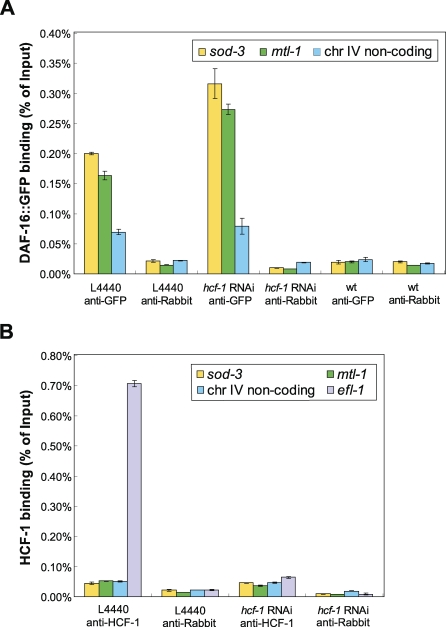
Loss of *hcf-1* Enhances the Enrichment of DAF-16 on the Promoters of Its Target Genes ChIP was performed using *daf-2(e1370);daf-16(mgDf47);daf-16::gfp* worms treated with control RNAi (L4440) or *hcf-1* RNAi. Synchronized adult worms of the different strains were incubated at 25 °C for ∼6 h to inactivate *daf-2* and to induce robust DAF-16::GFP nuclear localization. Worm extracts were subjected to immunoprecipitation using anti-GFP, anti-HCF-1, or anti-Rabbit IgG. The recovered DNA was quantitated using qPCR. Regions around the DAF-16 binding element (DBE) at the *sod-3* or *mtl-1* promoters, as well as a putative noncoding region of Chromosome IV not containing any DBE were monitored. The figure shows one representative experiment. Error bars represent the SEM of the duplicated reactions in qPCR. Similar results were obtained for three independent experiments. (A) DAF-16 enrichment at the promoters of *sod-3* or *mtl-1* was enhanced upon *hcf-1* RNAi. DAF-16 was robustly enriched at the *sod-3* or *mtl-1* promoters after anti-GFP ChIP compared to that of anti-Rb (anti-GFP/anti-Rabbit at *sod-3* promoter: ∼9-fold; anti-GFP/anti-Rabbit at *mtl-1* promoter: ∼11-fold). The fold enrichment of DAF-16 at *sod-3* or *mtl-1* was consistently greater than that at the nonspecific Chromosome IV region: *sod-3*/chr IV: ∼2.9-fold; *mtl-1*/chr IV: ∼2.4-fold. As a control, anti-GFP ChIP in wild-type (wt) worms (not expressing *daf-16::gfp*) showed background signal that was very similar to that of anti-Rabbit. Upon *hcf-1* RNAi knockdown, DAF-16 enrichment at the *sod-3* or *mtl-1* promoters was greatly increased. Anti-GFP/anti-Rabbit at *sod-3* promoter: ∼33-fold (versus ∼9-fold for L4440 RNAi); anti-GFP/anti-Rabbit at *mtl-1* promoter: ∼33-fold (versus ∼11-fold for L4440 RNAi). In contrast, for the nonspecific Chromosome IV region, anti-GFP/anti-Rabbit: ∼4-fold (versus ∼3-fold for L4440 RNAi). These data indicated that in the absence of HCF-1, a greater amount of DAF-16 becomes recruited to the *sod-3* or *mtl-1* promoters, but the nonspecific binding of DAF-16 to the Chromosome IV region is not substantially changed. (B) HCF-1 was greatly enriched at the *efl-1* promoter, but not at *sod-3* or *mtl-1* promoters. *efl-1* is the C. elegans homolog of *E2f1*, which has been shown to be a direct target of HCF-1 in mammalian cells [53]. The region of the *efl-1* promoter containing a conserved E2F1 binding element was included as a positive control for anti-HCF-1 ChIP. Whereas HCF-1 was found to be greatly enriched at the *efl-1* promoter, it was not substantially enriched at the promoters of *sod-3* or *mtl-1*, or the Chromosome IV noncoding region. Fold change of anti-HCF-1/anti-Rabbit at *efl-1*, *sod-3*, *mtl-1*, Chromosome IV noncoding region: ∼30.8, ∼2.1, ∼3.6, ∼2.3, respectively. As a control, when *hcf-1* was knocked down by RNAi, the enrichment of HCF-1 on the promoter of *efl-1* was greatly reduced.

To further elucidate the molecular mechanism by which HCF-1 regulates DAF-16-mediated transcription, we performed chromatin immunoprecipitation (ChIP) experiments to test how HCF-1 might affect DAF-16 enrichment on target gene promoters. For the ChIP experiments, we employed *daf-16::gfp* worms (*daf-2(e1370);daf-16(mgDf47);daf-16::gfp*) and anti-GFP immunoprecipitation to capture DAF-16. As previously reported [48], DAF-16 was enriched at the promoter of *sod-3* when the IIS pathway was inactivated (Figure 7A). We also examined the DAF-16 target gene *mtl-1* and found that DAF-16 was enriched on its promoter (Figure 7A). Under the same ChIP conditions, we did not detect HCF-1 enrichment at the *sod-3* or *mtl-1* promoters, but detected great enrichment of HCF-1 at the promoter of *efl-1* (Figure 7B). *efl-1* is the *C. elegans E2f1* gene; in mammalian cells, HCF-1 is highly enriched at the promoter region of *E2f1* [53]. Thus, our results demonstrate a conserved role of HCF-1 at the *efl-1*/*E2f1* promoter in worms, and suggest that HCF-1 is not likely to present at *sod-3* or *mtl-1* promoters. Considering our co-IP results showing that HCF-1 and DAF-16 physically associate in worms (Figure 6), our ChIP results suggest that the HCF-1/DAF-16 complex is probably not present at the promoters of DAF-16 target genes. However, it remains possible that HCF-1 is a component of a large protein complex that associates with DAF-16 at target gene promoters, and our cross-linking conditions cannot capture the presence of HCF-1 at those promoters. For the other *hcf-1*-regulated, *daf-16*-dependent genes (Table 4; *F21F3.3* and *C32H11.4*), we either did not identify a putative DAF-16 binding element or did not detect DAF-16 enrichment at the promoter regions we surveyed, suggesting that they might not be DAF-16 direct targets. We next tested whether the enrichment of DAF-16 on *sod-3* or *mtl-1* promoters would be affected when *hcf-1* was RNAi depleted. As a control, we showed that the enrichment of HCF-1 on the *efl-1* promoter was substantially reduced when *hcf-1* was knocked down by RNAi. Interestingly, under the same RNAi conditions, we consistently observed an enhanced enrichment of DAF-16 to the *sod-3* and *mtl-1* promoters in multiple independent trials (Figure 7A). These results suggest that in the absence of HCF-1, more DAF-16 is able to localize to the promoters of its target genes.

## Discussion

**Figure 8:**
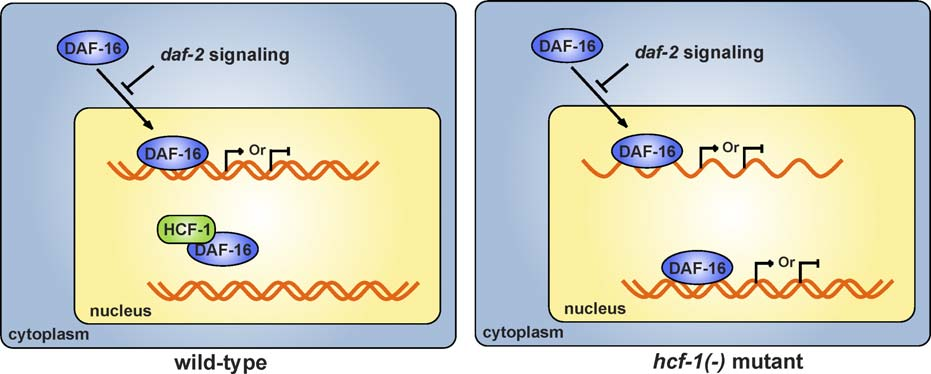
The Model We propose that in wild-type C. elegans, HCF-1 associates with a fraction of DAF-16 in the nucleus and limits the recruitment of some DAF-16 to its target gene promoters. Inactivation of *hcf-1* allows more DAF-16 to access its target gene promoters and enforces DAF-16-mediated regulation of selective target genes, which likely contributes to the prolonged lifespan and enhanced stress resistance phenotypes of the *hcf-1* mutants.

Our study has revealed the C. elegans homolog of HCF-1 to be an important longevity determinant and transcriptional regulator of DAF-16. Our data indicate that HCF-1 is necessary for maintaining normal lifespan and stress response in C. elegans, as loss of *hcf-1* results in mutant worms with substantially extended longevity and heightened resistance to specific stress stimuli. In modulating C. elegans lifespan and stress response, *hcf-1* completely depends on the activity of *daf-16*, but likely acts independently of the IIS pathway. In elucidating the mechanism by which HCF-1 regulates DAF-16, we showed that HCF-1 is a ubiquitously expressed nuclear protein and forms a protein complex with DAF-16 in C. elegans. Interestingly, in the absence of *hcf-1*, greater enrichment of DAF-16 at its target gene promoters is observed and more robust DAF-16-mediated regulation of selective transcriptional targets is detected. On the basis of our results, we propose that HCF-1 modulates C. elegans lifespan and stress response by acting as a novel negative regulator of DAF-16. Normally, HCF-1 associates with DAF-16 and limits a fraction of DAF-16 from accessing its target gene promoters. In the absence of HCF-1, more DAF-16 is released to localize to target gene promoters to confer greater transcriptional regulation of selective target genes (Figure 8). Altered expression of this subset of target genes likely contributes to the stress resistance and prolonged longevity phenotypes observed in the *hcf-1* mutant worms.

Our results ascribe a new longevity and stress response function to the highly conserved transcriptional regulator HCF-1. Mammalian HCF-1 was first identified as a major host cell factor required for HSV VP16-induced immediate early gene transcription [29]. In addition to its role in HSV infection, HCF-1 is also essential for cell cycle progression. Studies have revealed that mammalian HCF-1 is required for appropriate transition from G1 to S phase, and also proper progression of M phase and cytokinesis [31–33]. Importantly, mammalian and C. elegans HCF-1 share conserved functions. C. elegans HCF-1 is able to stabilize the VP16-induced complex; moreover, *C. elegans hcf-1* mutants produce small broods and exhibit low penetrance of embryonic lethality (Figure S2) [35], both phenotypes consistent with a role of HCF-1 in cell proliferation. Furthermore, *C. elegans hcf-1* mutant embryos show low penetrance of mitotic and cytokinetic defects [35]. As HSV is a human specific virus, it is thought that VP16 likely mimicked a cellular interaction with HCF-1 and co-opted human HCF-1 for productive HSV lytic infection. Given the well-established and conserved role of HCF-1 in cell cycle control, it is interesting to consider whether the cell proliferation function of HCF-1 is linked to its role in longevity and stress response. In adult C. elegans, the only proliferative tissue is the germline. Whereas a defect in germline stem cell proliferation is known to cause lifespan increase, our genetic studies showed that *hcf-1* deficiency can continue to extend the lifespan of worms completely lacking germline (Figure 1F; Table 1), suggesting that the longevity function of *hcf-1* is likely not linked to its role in cell proliferation. The high degree of functional conservation between C. elegans and mammalian HCF-1 suggests that mammalian HCF-1 likely also participates in stress response and longevity determination. Therefore, HCF-1 may very well represent a new universal longevity determinant.

Our model proposes that HCF-1 affects lifespan and stress response by forming a protein complex with DAF-16 and regulating DAF-16 recruitment to target gene promoters and DAF-16-mediated gene transcription. This model is consistent with the known roles of mammalian HCF-1. For VP16-induced immediate early (IE) gene expression, binding of HCF-1 to VP16 is thought to help recruit the activating Set1/Ash2 histone methyltransferase complex to IE-gene promoters [29]. For its role in G1/S transition, mammalian HCF-1 has been found to recruit the Set1/Ash2 histone methyltransferase activating complex to E2F1 and the Sin3 histone deacetylase repressive complex to E2F4 at the appropriate times of the cell cycle, which likely helps to reinforce the activating or repressive functions of the respective E2F family members [33]. The role of HCF-1 in regulating the transcription factor Miz-1 is particularly relevant to this study. HCF-1 has been shown to physically associate with Miz-1 and antagonize Miz-1-mediated transactivation by interfering with the association of Miz-1 and the histone deacetylase P300 [49]. Furthermore, HCF-1, via its various functional motifs, has been shown to mediate protein–protein interactions with a large number of polypeptides, including transcription factors LZIP/Luman, Zhangfei, HPIP, Sp1, and GABPβ, protein phosphatase PP1, and cell-death protein PDCD2 [29]. HCF-1 is emerging as an extremely versatile scaffolding protein, capable of binding to many different transcription and chromatin factors via its different conserved motifs, and assembling appropriate protein complexes for proper context-dependent gene expression regulation [33,49–51].

Our qRT-PCR results suggest that HCF-1 is only able to regulate the DAF-16-mediated transcription of a select group of previously identified DAF-16 target genes. Considering that the DAF-16 target genes we surveyed were previously determined to be responsive to *daf-2/IIS*, and since our genetic studies suggest that *hcf-1* and *daf-2* might act in parallel pathways and converge onto *daf-16* (Figure 1), it is not surprising that some of the gene expression changes caused by *hcf-1* deficiency would be distinct from that caused by reduced *daf-2* signaling. It can be argued that HCF-1 might represent a weaker regulator of DAF-16 compared to DAF-2, and its effect on some of the DAF-16-regulated genes might simply be missed in our analysis as it is likely to be much weaker than that in the *daf-2* mutant (Table 3). Whereas a weaker effect model is possible, we favor the model that HCF-1 represents a gene-specific regulator of DAF-16. In our analysis, we noticed that the impact of *hcf-1* deficiency on DAF-16 target gene regulation is not always much weaker than reduced *daf-2* signaling. For instance, the expression fold change of *F21F3.3* in *hcf-1(-)* was comparable with that in *daf-2(-)* (∼5-fold versus ∼11-fold). On the other hand, there are genes, e.g., *dod-3* and *dod-24*, whose expression change in *daf-2(-)* is as great as that of *sod-3* or *mtl-1* (∼100-fold), however, unlike *sod-3* and *mtl-1*, their gene expression did not show a significant change in *hcf-1(-).* Future whole genome expression profiling experiments will provide a global view of whether HCF-1 acts as a gene-specific negative regulator of DAF-16. Considering that HCF-1 and DAF-16/FOXO are highly conserved across species, it is very likely that mammalian HCF-1 also conserves the function of FOXO regulation. Whereas C. elegans only has one *daf-16* gene, mammals have four FOXO genes. Context specific regulators of FOXOs are critical in ensuring specificity on gene expression regulation and subsequent biological responses [14]. Our findings in C. elegans raise the intriguing possibility that mammalian HCF-1 represents a new regulator of one or more of the FOXO proteins.

An important next question is how HCF-1 might achieve its specificity in influencing DAF-16 transcriptional targets. A simple hypothesis is that the association between HCF-1 and DAF-16 may be regulated on the basis of upstream stimulus. For example, signals that induce altered subcellular localization of HCF-1 or DAF-16 may disrupt the HCF-1/DAF-16 interaction. Both HCF-1 and DAF-16 has been shown to shuttle between the nucleus and cytoplasm under specific conditions [14,37,54–56]. In addition, DAF-16/FOXO and HCF-1 proteins have been shown to be extensively modified post-translationally [14,37,52,57–59]. It is possible that different modifications on HCF-1 and/or DAF-16 will substantially affect their association. Lastly, the specificity toward target genes can also be conferred at the level of the transcriptional complex assembly. Under this scenario, additional co-regulators of DAF-16 will likely come into play. Whereas HCF-1 represents the only nuclear negative regulator of DAF-16 known in C. elegans, several DAF-16 nuclear positive regulators have been reported, including SIR-2.1 [24], SMK-1 [27], and BAR-1 [28]. Future research to elucidate the interplay among the various DAF-16 co-activators and HCF-1 will greatly advance our understanding of the mechanistic details of how DAF-16 transcriptional activities can be appropriately regulated.

Our data indicate that an important function of HCF-1 may be to modulate responses to specific environmental stress stimuli. Interestingly, only the *hcf-1(pk924)* allele showed resistance to paraquat and cadmium treatment, and neither of the *hcf-1* mutant alleles demonstrated altered response to heat shock. These results suggest that a general heightened response to a wide-range of environmental stresses is not likely to account for the lifespan increase observed in the *hcf-1* mutant worms. However, it is important to also consider that in the stress assays, worms were challenged with a high dose of an acute stress, which is very different from the low level of chronic stress worms experience as they grow old in longitudinal assays. The involvement of *hcf-1* in stress response nicely fits with the overall theme that HCF-1 is a gene-specific transcriptional regulator of DAF-16. It is well established that distinct stress stimuli are able to induce DAF-16/FOXO to regulate different target genes [13,14]. We propose that the main role of HCF-1 is to help fine tune the regulation of a subset of DAF-16-regulated genes to modulate survival under specific conditions. Taken together, we showed that HCF-1 is essential for longevity maintenance and that it functions as a negative regulator of DAF-16 in C. elegans. As HCF-1 and DAF-16/FOXO are highly conserved from C. elegans to mammals, our findings have important implications for FOXO regulation and longevity determination in diverse organisms.

## Materials and Methods

### C. elegans strains.

The strains used in this paper were as follow: wild-type N2, *daf-16 (mgDf47)*, *daf-2 (e1370)*, *age-1(hx546)*, *sqt-1(sc13) age-1(mg44)/mnC1* (a gift from Catherine A. Wolkow, National Institute of Aging), *glp-1(e2141), hcf-1(ok559)* (generated by the C. elegans Gene Knockout Consortium), *hcf-1(pk924)* (a gift from Winship Herr, University of Lausanne, Switzerland), *daf-16(mgDf47);xrIs87[daf-16α::gfp::daf-16b, rol-6(su1006)]* (DAF-16::GFP) [52], *daf-16(mu86);muIs71[daf-16a::gfp/bKO*, *rol-6(su1006)*] (DAF-16::GFP) [37], and *muIs84[Psod-3::gfp]* [60]. The *hcf-1(ok559)* allele was outcrossed five times and the *hcf-1(pk924)* allele was outcrossed three times with the N2 strain in our lab prior to phenotype analyses.

The following double mutant strains were constructed using standard genetic methods: *daf-16(mgDf47);hcf-1(ok559), daf-16(mgDf47);hcf-1(pk924), daf-2(e1370);hcf-1(ok559), daf-2(e1370);hcf-1(pk924), sqt-1(sc13) age-1(mg44)/mnC1;hcf-1(ok559),glp-1(e2141);hcf-1(pk924), daf-16(mu86);muIs71[daf-16a::gfp/bKO, rol-6(su1006)];hcf-1(ok559), daf-16(mu86);muIs71[daf-16a::gfp/bKO* and *hcf-1(pk924);sur-5::gfp, muIs84[Psod-3::gfp];hcf-1(pk924)*.

All strains were cultured using standard methods [61]. Unless otherwise stated, NGM plates were seeded with E. coli OP50 as the food source.

### Lifespan assays.

For RNAi lifespan assays, RNAi bacteria were grown in Luria broth with 50 μg/ml ampicillin at 37 °C for 10–16 h, seeded onto NGM plates containing 2 or 4 mM IPTG, and induced at room temperature for about 6 h [62]. Worms were allowed to lay eggs overnight on RNAi plates at 16 °C, and the progeny were grown on RNAi plates at 25 °C until they developed into young adult stage. The young adult worms were then transferred onto RNAi plates seeded with 3-fold concentrated RNAi bacteria that contained 50 μg/ml FUDR to prevent the growth of progeny. For lifespan assays using NGM plates seeded with OP50 bacteria, worms were allowed to lay eggs over-night at 16 °C, and the progeny were grown on NGM plates at 25 °C till young adult stage. The young adult worms were then transferred onto NGM plates that contained 50 μg/ml FUDR and seeded with 3-fold concentrated E. coli OP50. For lifespan assays involving *daf-2(e1370)* and *daf-2(e1370);hcf-1(pk924)* or *daf-2(e1370);hcf-1(ok559)* worms, progeny were allowed to grow at 16 °C and shifted to 25 °C after the L3 larval stage to avoid the constitutive dauer arrest phenotype associated with the *daf-2(e1370)* mutant [63].

For all lifespan assays, worms were aged at 25 °C. Worms were scored every day or every other day, and those that failed to respond to a gentle prodding with a platinum wire were scored as dead. Animals that bagged, exploded, or crawled off the plate were considered as censored. We defined the day when we transferred the young adult worms as day 0 of adult lifespan. Statistical analysis was done using the SPSS software and *p*-values were calculated using the log-rank test. All the lifespan experiments were repeated at least two independent times.

### Transgenic animals.

A GFP-fused *hcf-1* plasmid (*Phcf-1*::*hcf-1::gfp*) was created by inserting a genomic fragment that contains ∼500 bp upstream of the predicted ATG of *hcf-1* and the entire predicted coding region of *hcf-1* into the pPD95_77 plasmid. Transgenic worms were created by microparticle bombardment as previously described [64]. The *Phcf-1::hcf-1::gfp* plasmid was co-bombarded with the pJKL702 [*unc-119(+)*] plasmid into the *unc-119(ed4)* mutant worms to obtain the strain *unc-119(ed4);rwIs3[Phcf-1::hcf-1::gfp, unc-119]*. Multiple independent integrated transgenic lines were examined.

### Embryonic and brood size assays.

Each single L4 worm was allowed to lay eggs and transferred to a fresh NGM plate every 24 h until it completed egglay. The number of eggs laid and the number of hatched worms were counted. A total of five worms were used for each strain.

### Nile red staining.

Nile red staining was performed as previously described [47]. Unseeded NGM plates were coated with 0.025 μg/ml final concentration Nile red that had been resuspended in acetone and diluted in H_2_O. The Nile red was allowed to diffuse through NGM overnight. The plates were then seeded with OP50 bacteria and left at room temperature overnight. Gravid adult worms were allowed to egglay onto the plates overnight, and progeny allowed to develop to young adult stage. Worms were then monitored using a fluorescent microscope (Leica DM 5000B) and images were captured using a Hamamatsu ORCA-ER camera and the OpenLab Software.

### Stress assays.

All stress assays were performed as previously described [41–43].

For the paraquat assay, gravid adult worms of each strain tested were allowed to lay egg on NGM plates seeded with OP50 for 2–3 h to produce relatively synchronous populations of progeny. The progeny were allowed to develop at 25 °C, and when they reached young-adulthood, FUDR was added to the plates at a final concentration of 50 μg/ml to prevent the growth of progeny. At day 2–3 of adulthood, worms were washed off the NGM plates and rinsed with M9 buffer three times to remove the OP50 bacteria. Approximately 30 adults were dispensed into each well of a 24-well culture plate containing 300 μl of 200 mM paraquat (Sigma) in M9 buffer. Triplicate wells were used for each strain, and the experiment was repeated at least two independent times. Worms in the paraquat buffer were scored every 2–3 h for survival. Worms that failed to respond to gentle prodding were scored as dead.

For the CdCl_2_ assay, synchronized day 2 adult worms were collected as described above and washed by K-medium. Worms were then put into each well of a 24-well culture plate containing 600 μl K-medium with 18 mM CdCl_2_ at 20 °C. Triplicate plates for each strain were scored for each time point indicated. Worms that failed to respond to gentle prodding were scored as dead.

For the heat shock assay, synchronous populations of worms were grown as described above. Day 2 adult worms grown on OP50-NGM plates were shifted to 35 °C. Duplicate or triplicate plates for each strain were scored for each time point. Because the scoring was done at room temperature, once the worms were pulled from 35 °C and scored for survival, they were discarded to avoid the complication of recovery from heat shock during the time of scoring.

### Dauer assays.

Dauer assays were performed as previously described [46]. Worms at the L4 stage were allowed to lay egg at16 °C over night. The resulting progeny were allowed to develop at the indicated temperature. At ∼96 h (25 °C or 27 °C) or ∼120 h (22 °C) after egg lay, the number of dauers and adult worms on each plate were scored. Replica plates were scored for each strain. The dauer assays were repeated two to three times.

### DAF-16::GFP localization assay.

*daf-16(mgDf47);xrls87* transgenic strain was used for DAF-16::GFP localization assay [52]. Worms at the L4 larval stage were picked onto RNAi plates and allowed to lay egg at 16 °C for one day. Progenies were exposed to the RNAi bacteria at 16 °C for 5 d until they became gravid adults. The DAF-16::GFP signal was then monitored using a fluorescent microscope (Leica DM 5000B), and images were captured using a Hamamatsu ORCA-ER camera and the OpenLab program.

### Immunostaining.

Worm fixation and immunostaining were performed as previously described [65]. In brief, worms were immobilized and compressed between two polylysine coated slides and snap frozen in liquid nitrogen. The cuticle was removed by quickly separating two frozen slides. Worms were then quickly fixed in pre-chilled methanol at −20 °C. Incubation with primary and secondary antibodies and washes were done in TBS buffer at room temperature. Fluorescence signal was monitored using a fluorescent microscope (Leica DM 5000B), and images were captured using a Hamamatsu ORCA-ER camera and the OpenLab program.

### Immunoprecipitation and immunoblotting.

Immunoprecipitation and immunoblotting were carried out as described [66]. In brief, for immunoprecipitation experiments, worm extracts were made from mixed staged worms by sonication of worms in lysis buffer and subsequent removal of debris by centrifugation. The appropriate antibody was incubated with worm extract at 4 °C overnight followed by incubation with protein A slurry (Pierce) at 4 °C for 3∼6 hrs. The protein A beads were then washed with lysis buffer for 6 times. The bound proteins were eluted by boiling in 2× sample buffer, separated on a SDS gel, transferred onto a nitrocellulose membrane, and followed by standard ECL detection.

### Antibodies.

To generate polyclonal antiserum against C. elegans HCF-1, bacterial recombinant S-tagged fusion protein containing full-length HCF-1 was purified using the S-Tag Thrombin Purification kit (Novagen) and used as an antigen to immunize guinea pigs. The crude anti-HCF-1 antiserum was subsequently purified using S-tagged HCF-1. In brief, the crude anti-HCF-1 antiserum was incubated with purified S-tagged HCF-1 immobilized on nitrocellulose membrane (BA85 Protran BioScience). Poorly bound proteins were removed by multiple washes in TBS and PBS buffer, and the bound anti-HCF-1 antibody was recovered by subsequent elution.

For immunostaining, affinity purified anti-HCF-1 antibody was used as the primary antibody (1:500) and anti-guinea pig conjugated with Cy3 (Jackson ImmunoResearch Laboratories, 1:200) was used as the secondary antibody. For GFP immunostaining, anti-GFP (goat, Rockland) was used as the primary antibody (1:1000) and anti-goat conjugated with FITC (Jackson ImmunoResearch Laboratories, 1:400) was used as the secondary antibody.

For immunoprecipitation, affinity purified anti-HCF-1 and anti-GFP (rabbit, Clontech) antibodies were used.

Antibodies for immunoblotting include: anti-DAF-16 (cC-20) (goat, Santa Cruz), anti-HCF-1, anti-GFP (rabbit, Clontech), anti-ACTIN (mouse, Chemicon), anti-goat (Rockland), anti-mouse (Santa Cruz), anti-guinea pig (Jackson ImmunoResearch Laboratories), and anti-rabbit (Rockland).

### RNA isolation and qRT-PCR.

Synchronized late L4 staged worms were used for RNA isolation. All the worms for qRT-PCR were grown at 25 °C except that in the experiments using *daf-2(e1370)* mutants, worms were shifted from 16 °C to 25 °C for 8 h prior to harvest. Total RNA from ∼10–15 μl of worm pellet was isolated using Tri-reagent (Molecular Research Center, Inc.) [67]. cDNAs were synthesized with random hexamers using SuperScript III First-Strand Kit (Invitrogen). qRT-PCR reactions were performed using iQ SYBR Green Supermix (BIO-RAD) and the MyiQ Single Color Real-time PCR Detection System (BIO-RAD). The qRT-PCR conditions were: 95 °C for 3 min, followed by a 40-cycles of 10 s at 95 °C and 30 s at 60 °C. Melting curve analysis was performed for each primer set at the end to ensure the specificity of the amplified product. For qRT-PCR, *act-1* was used as the internal control, and the RNA level of each gene of interest was normalized to the level of *act-1* for comparison. The qRT-PCR experiment was repeated at least three times using independent RNA/cDNA preparations. The data were pooled and analyzed using Student's *t*-test.

### qRT-PCR primers.

The qRT-PCR primers for *act-1* are: forward primer: 5′-CCAGGAATTGCTGATCGTATGCAGAA-3′; reverse primer: 5′-TGGAGAGGGAAGCGAGGATAGA-3′ (product length: 133 bp). Primers for *sod-3* are: forward primer: 5′-CCAACCAGCGCTGAAATTCAATGG-3′; reverse primer: 5′-GGAACCGAAGTCGCGCTTAATAGT-3′ (product length: 127 bp). Primers for *mtl-1* are: forward primer: 5′-atggcttgcaagtgtgactg-3′; reverse primer: 5′-cacatttgtctccgcacttg-3′ (product length: 56 bp). Primers for *fat-5* are: forward primer: 5′-tggtgaagaagcacgatcag-3′; reverse primer: 5′-aagcagaagattccgaccaa-3′ (product length: 125 bp). Primers for M02D8.4 are: forward primer: 5′-atttgccaacaaacatgcaa-3′; reverse primer: 5′-ggtccacgatggtgttgtct-3′ (product length: 87 bp). Primers for *ges-1* are: forward primer: 5′-agcaacaaggaagggtcgta-3′; reverse primer: 5′-ccgatgatctccgaaatgaa-3′ (product length: 120 bp). Primers for *lys-7* are: forward primer: 5′-gcgggttattgtgcagtttt-3′; reverse primer: 5′-tcaattccgagtccagcttt-3′ (product length: 114 bp). Primers for K09C4.5 are: forward primer: 5′-tggaattgaaccgactattgc-3′; reverse primer: 5′-gcaaatggcacaagaacaaa-3′ (product length: 106 bp). Primers for *dod-3* are: forward primer: 5′-AAAAAGCCATGTTCCCGAAT-3′; reverse primer: 5′-GCTGCGAAAAGCAAGAAAAT-3′ (product length: 137 bp). Primers for F21F3.3 are: forward primer: 5′-CCGATTCGTTCCTTTTGAAG-3′; reverse primer: 5′-ACAACCGAATGTTCCAATCC-3′ (product length: 138 bp). Primers for *hsp-16.1* are: forward primer: 5′-GCAGAGGCTCTCCATCTGAA-3′; reverse primer: 5′-GCTTGAACTGCGAGACATTG-3′ (product length: 85 bp). Primers for *hsp-12.3* are: forward primer: 5′-GCCATTCCAGAAAGGAGATG-3′; reverse primer: 5′-CGTTTGGCAAGAAGTTGTGA-3′ (product length: 93 bp). Primers for C32H11.4 are: forward primer: 5′-TTACTTCCCATCGCCAAAGT-3′; reverse primer: 5′-CAATTCCGGCGATGTATGAT-3′ (product length: 117 bp). Primers for F35E12.5 are: forward primer: 5′-TCTCGAAGCCAACAAGTTCA-3′; reverse primer: 5′-TTTCACGGGATCCGTATTTC-3′ (product length: 78 bp). Primers for T16G12.1 are: forward primer: 5′-CAATGGGAGCTCACTTCGAT-3′; reverse primer: 5′-TCATCGGCAAGAAGAGTCAA-3′ (product length: 138 bp). Primers for *dod-24* are: forward primer: 5′-TGTCCAACACAACCTGCATT-3′; reverse primer: 5′-TGTGTCCCGAGTAACAACCA-3′ (product length: 138 bp). Primers for F08G5.6 are: forward primer: 5′-tggacaacccagatatgcaa-3′; reverse primer: 5′-gtatgcgatggaaatggaca-3′ (product length: 111 bp). Primers for *dod-22* are: forward primer: 5′-ttgttggtcccaagttcaca-3′; reverse primer: 5′-aagaacttcggctgcttcag-3′ (product length: 132 bp). Primers for *daf-16* are: forward primer: 5′-ccagacggaaggcttaaact-3′; reverse primer: 5′-attcgcatgaaacgagaatg–3′ (product length: 149 bp). Primers for *daf-2* are: forward primer: 5′-cggtgcgaagagaggatatt-′; reverse primer: 5′-tacagaggtcgccgttactg-3′ (product length: 97 bp). Primers for *age-1* are: forward primer: 5′-agtggattcggaaacaatgc-3′; reverse primer: 5′-ggaatcgatcgacactttca-3′ (product length: 135 bp). Primers for *akt-1* are: forward primer: 5′-tcaccgatgcgatattgtct-3′; reverse primer: 5′-aactccccaccaatcaacac-3′ (product length: 82 bp).

### ChIP.

ChIP was performed as previously described with slight modifications (P. Kolasinska-Zwierz, I. Latorre, and J. Ahringer, personal correspondence) [48]. In brief, ground frozen worm powder was crosslinked using 1% formaldehyde in PBS buffer and subjected to sonication. Immunoprecipitation was performed as described above. The protein-DNA complexes were then eluted from protein A beads and treated with RNase A and proteinase K. Precipitated DNA fragments were purified and subjected to qPCR analysis.

### qPCR primers for ChIP.

The qPCR Primers for ChIP are as followed. Primers for *sod-3* are: forward primer: 5′-TTTTCAAACCGAAAATTGACC-3′; reverse primer: 5′-CAAAGACCTCATCAACAGCAA-3′. Primers for *mtl-1* are: forward primer: 5′-ggccaccctcttttatcaca-3′; reverse primer: 5′-tcaaattgagctgccttcttc-3′. Primers for *efl-1*are: forward primer: 5′-ttttatcttctcattcaagcgaaa-3′; reverse primer: 5′-gagacaatgggaaaggtgga-3′. Primers for Chromosome IV noncoding region are: forward primer: 5′-CTCTTCATTTTGTTCCTGTGTTTTCC-3′; reverse primer: 5′-GAAGGCGGCGGTAATTGTTG-3′.

## Supporting Information

Figure S1 The *hcf-1(ok559)* Mutant Shows Weak Expression of a Truncated HCF-1 Protein and the *hcf-1(pk924)* Mutant Shows No Detectable Expression of Any HCF-1 Peptide Low levels of a truncated HCF-1 protein in the *hcf-1(ok559)* mutant were detected in immunoblotting assays using an affinity-purified polyclonal HCF-1 antibody generated against a full-length HCF-1 fusion protein. No partial HCF-1 protein was detected in the immunoblotting assays, suggesting that *hcf-1(pk924)* may represent a null mutant. Total protein from mixed populations of worms was separated on 15% SDS gel and followed by immunoblotting with an affinity-purified HCF-1 antibody. Actin level was used as a loading control.
(103 KB TIF)

Figure S2 The *hcf-1(ok559)* Mutant Shows Reduced Brood Size and Increased Embryonic Lethality The brood size and embryonic lethality were obtained from average of five animals for each group under 16 °C, 20 °C, and 25 °C. Error bars indicate standard error of the means.
(350 KB JPG)

Figure S3 GFP-Fused HCF-1 Is Expressed in the Nucleus of Somatic and Germline Cells (A) The images show the GFP expression of live transgenic worms carrying low-copy number of the *hcf-1::gfp* transgene (*rwIs3[Phcf-1::hcf-1::gfp, unc-119]*) at different developmental stages under normal culture condition. HCF-1::GFP is expressed in the nucleus of somatic and germline cells. Arrowhead indicates the high levels of autofluorescence observed in the intestine of the adult worm (bottom left panel).
(B) *hcf-1::gfp* worms were fixed and immunostained using anti-GFP. HCF-1::GFP is expressed in the nucleus of somatic (upper panel) and germline cells (bottom panel). DAPI staining was used to indicate the nucleus. Photos were taken at 400× magnification. Arrowhead indicates the autofluorescence observed in the intestine.
(2.26 MB TIF)

Figure S4 Loss of *hcf-1* Does Not Affect Fat Storage Fat storage in *hcf-1(pk924)* and wild-type worms were monitored by staining with the vital dye Nile Red [47]. Nile Red staining pattern of *hcf-1(pk924)* was similar to that in wild-type worms.
(642 KB TIF)

Figure S5 *Psod-3::gfp* Expression Level Is Elevated in *hcf-1(pk924)* Mutants The GFP levels of *Psod-3::gfp* (*muIs84[Psod-3::gfp]*) in *hcf-1(pk924)* mutant was elevated (right panel) compared to that in wild-type background (left panel). Synchronized day 2 adults were shown in the photos. Arrowhead indicates the intestinal autofluorescence.
(1.20 MB TIF)

Figure S6 HCF-1 Co-Localizes with DAF-16::GFP in the Nucleus Transgenic worms over-expressing DAF-16::GFP (*daf-16(mgDf47);xrIs87*) were immunostained with anti-HCF-1. Photos were taken at 100× (A) or 400× (B) magnification and representative images are shown. Under normal culturing condition, DAF-16::GFP was diffusely localized in the cytoplasm and nucleus as previously reported [37,52]. HCF-1 co-localized with DAF-16::GFP in the nucleus of nongermline cells. DAPI staining was used to indicate the nucleus. Arrowheads indicate the nucleus of intestinal cells. Arrows indicate the gonad of the worms where DAF-16::GFP expression is absent as a result of transgene silencing [68].
(1.70 MB TIF)

Table S1 Inactivation of *hcf-1* Results in Lifespan Increase That Is Completely Dependent on *daf-16*, but Likely Independent of the IIS Pathway (53 KB DOC)

Table S2 Expression of a *hcf-1::gfp* Transgene Partially Rescues the Lifespan Phenotype of *hcf-1(pk924)*
(33 MB DOC)

### Accession numbers

The wormbase (WB, http://www.wormbase.org) gene ID for *hcf-1* is WBGene00001827.

## Abbreviations

ChIP: chromatin immunoprecipitation
co-IP: co-immunoprecipitation
FOXO: forkhead box O transcription factor
GFP: green fluorescent protein
HCF-1: host cell factor 1
HSV: herpes simplex virus
IGF: insulin-like growth factor
IIS: insulin/IGF signaling
PI3K: phosphoinositide 3-kinase
qRT-PCR: quantitative reverse transcription PCR
RNAi: RNA interference
