# Supplementary figures and images for: Caenorhabditis elegans HCF-1 Functions in Longevity Maintenance as a DAF-16 Regulator
Source: PLoS Biol. 2008 Sep 30;6(9):e233. doi: 10.1371/journal.pbio.0060233 (PMC2553839; doi:10.1371/journal.pbio.0060233)

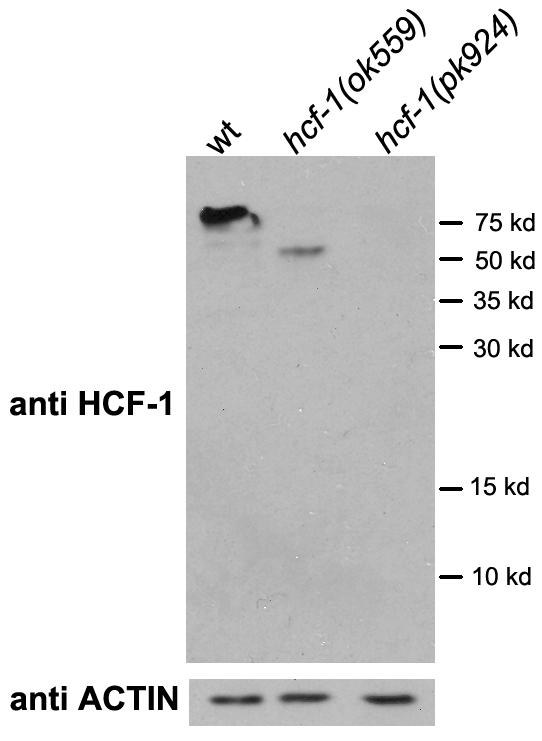

Supplement: Figure S1 — Low levels of a truncated HCF-1 protein in the hcf-1(ok559) mutant were detected in immunoblotting assays using an affinity-purified polyclonal HCF-1 antibody generated against a full-length HCF-1 fusion protein. No partial HCF-1 protein was detected in the immunoblotting assays, suggesting that hcf-1(pk924) may represent a null mutant. Total protein from mixed populations of worms was separated on 15% SDS gel and followed by immunoblotting with an affinity-purified HCF-1 antibody. Actin level was used as a loading control. (103 KB TIF) [file pbio.0060233.sg001.tif]

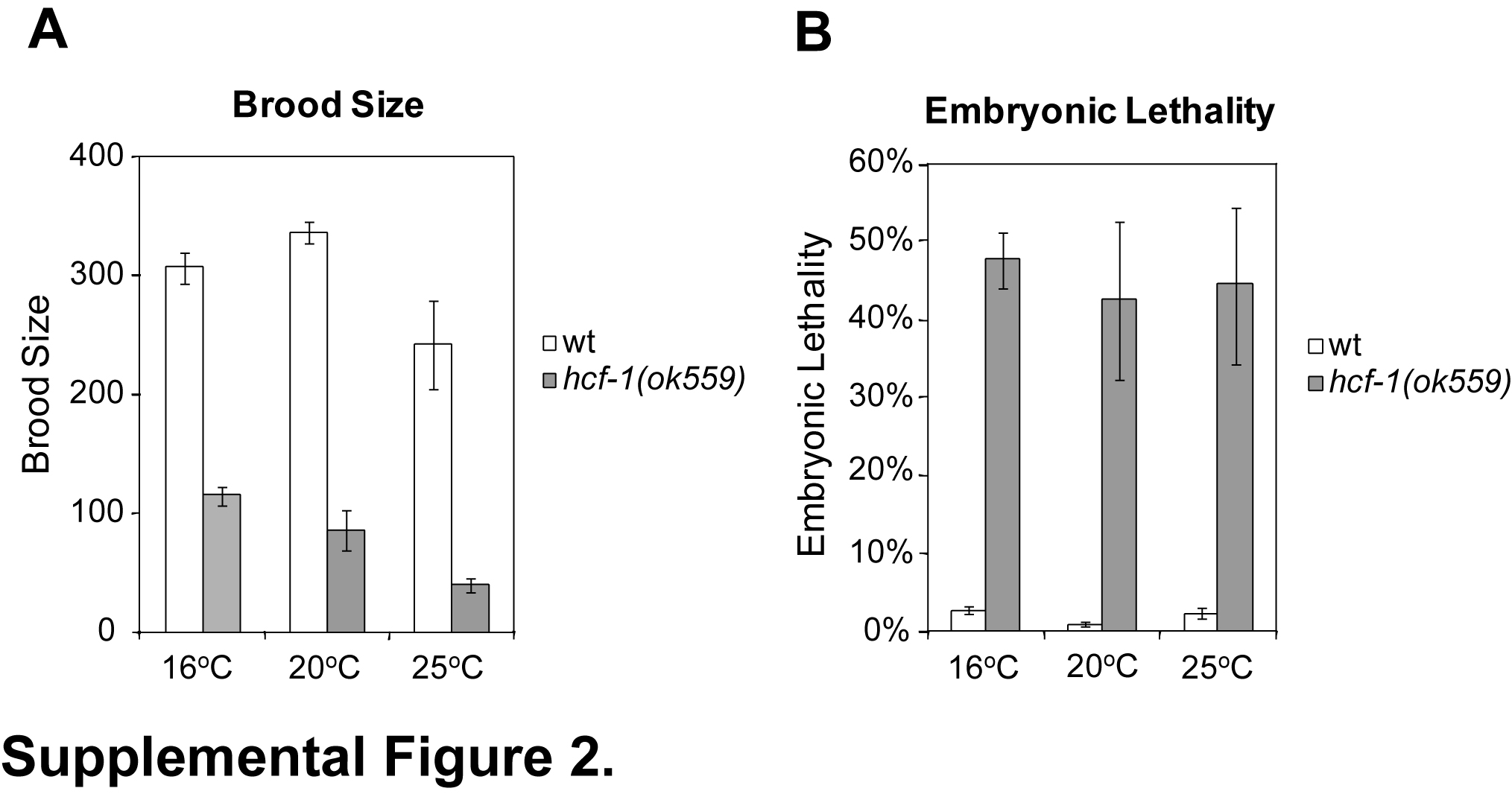

Supplement: Figure S2 — The brood size and embryonic lethality were obtained from average of five animals for each group under 16 °C, 20 °C, and 25 °C. Error bars indicate standard error of the means. (350 KB JPG) [file pbio.0060233.sg002.jpg]

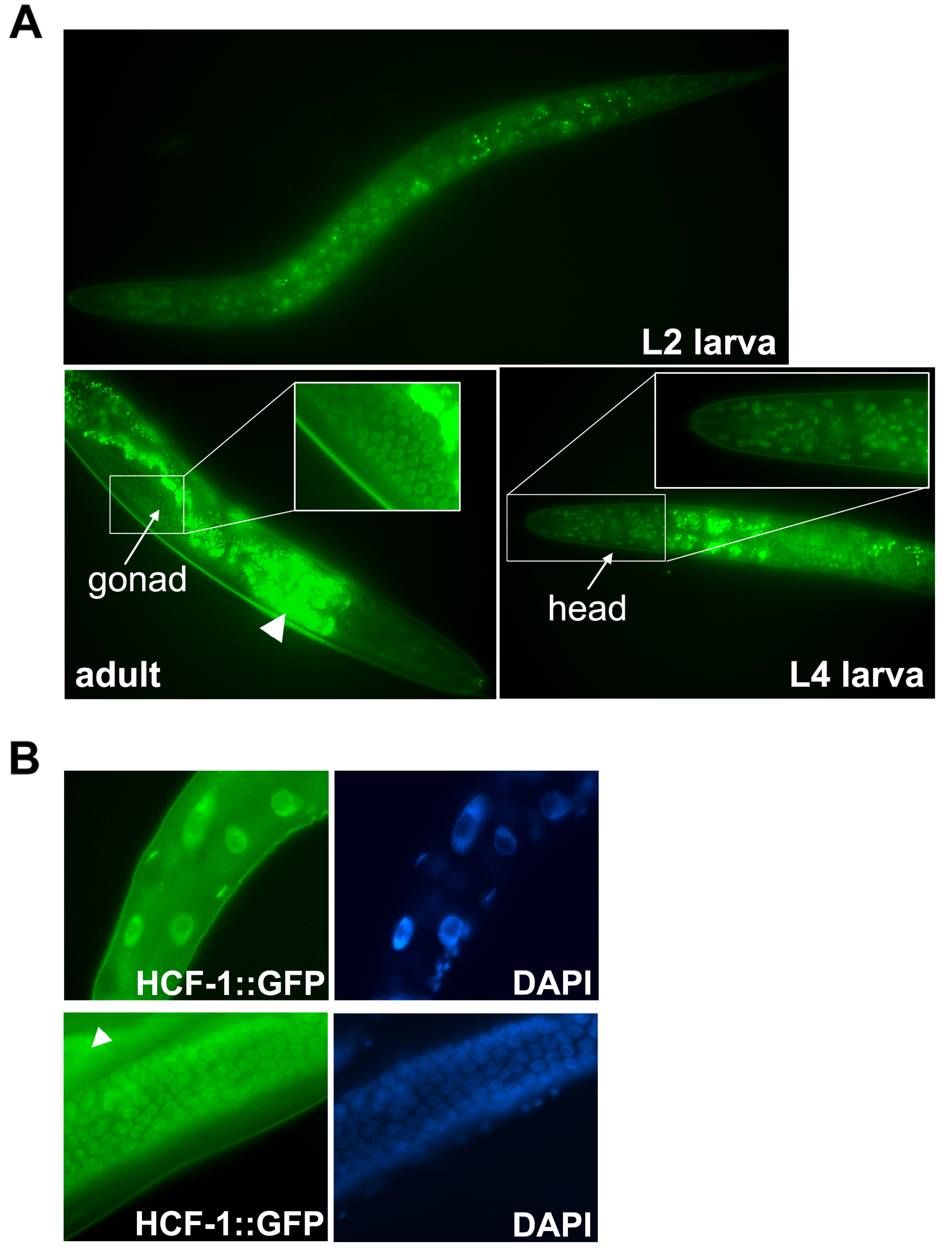

Supplement: Figure S3 — (A) The images show the GFP expression of live transgenic worms carrying low-copy number of the hcf-1::gfp transgene (rwIs3[Phcf-1::hcf-1::gfp, unc-119]) at different developmental stages under normal culture condition. HCF-1::GFP is expressed in the nucleus of somatic and germline cells. Arrowhead indicates the high levels of autofluorescence observed in the intestine of the adult worm (bottom left panel). (B) hcf-1::gfp worms were fixed and immunostained using anti-GFP. HCF-1::GFP is expressed in the nucleus of somatic (upper panel) and germline cells (bottom panel). DAPI staining was used to indicate the nucleus. Photos were taken at 400× magnification. Arrowhead indicates the autofluorescence observed in the intestine. (2.26 MB TIF) [file pbio.0060233.sg003.tif]

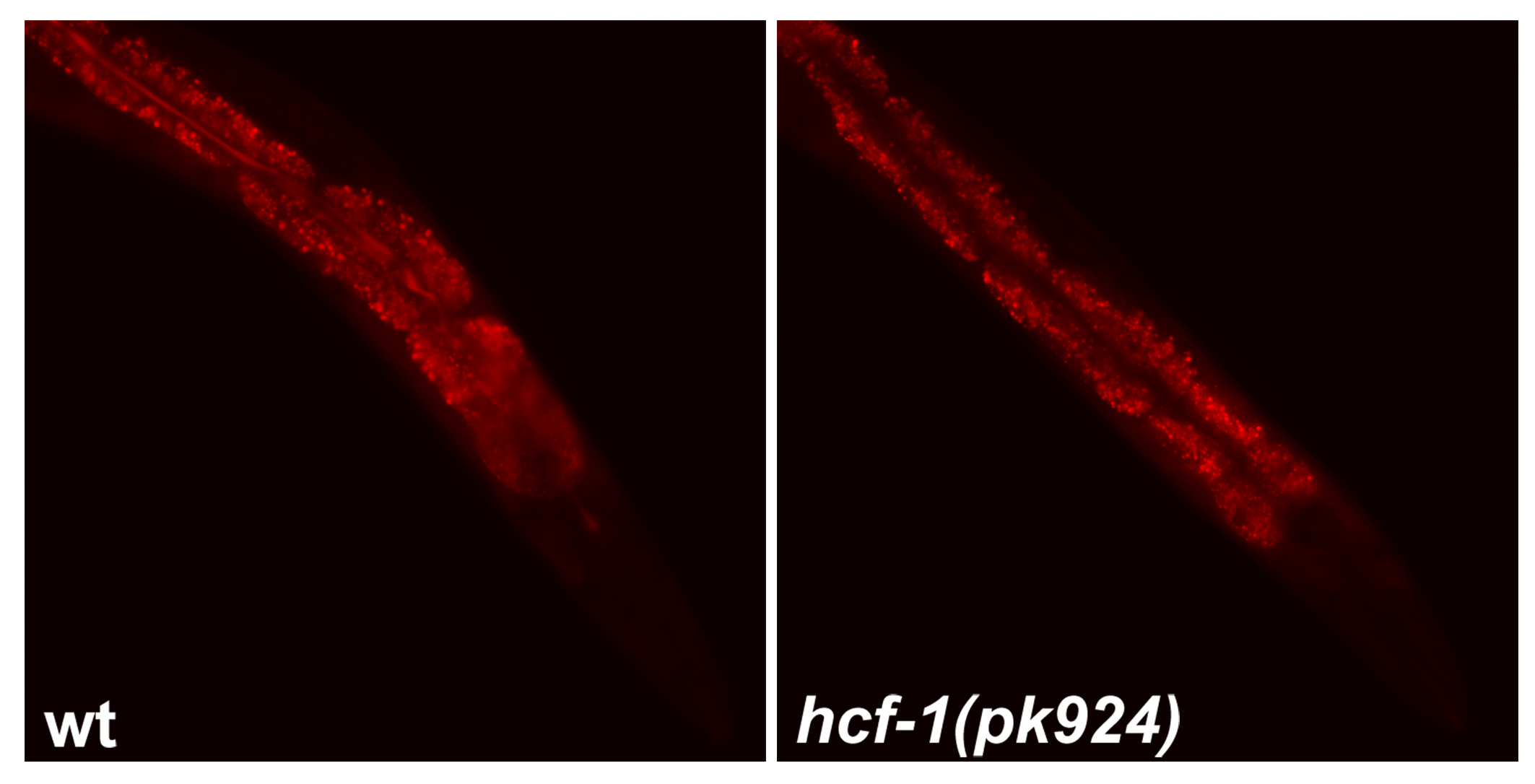

Supplement: Figure S4 — Fat storage in hcf-1(pk924) and wild-type worms were monitored by staining with the vital dye Nile Red [47]. Nile Red staining pattern of hcf-1(pk924) was similar to that in wild-type worms. (642 KB TIF) [file pbio.0060233.sg004.tif]

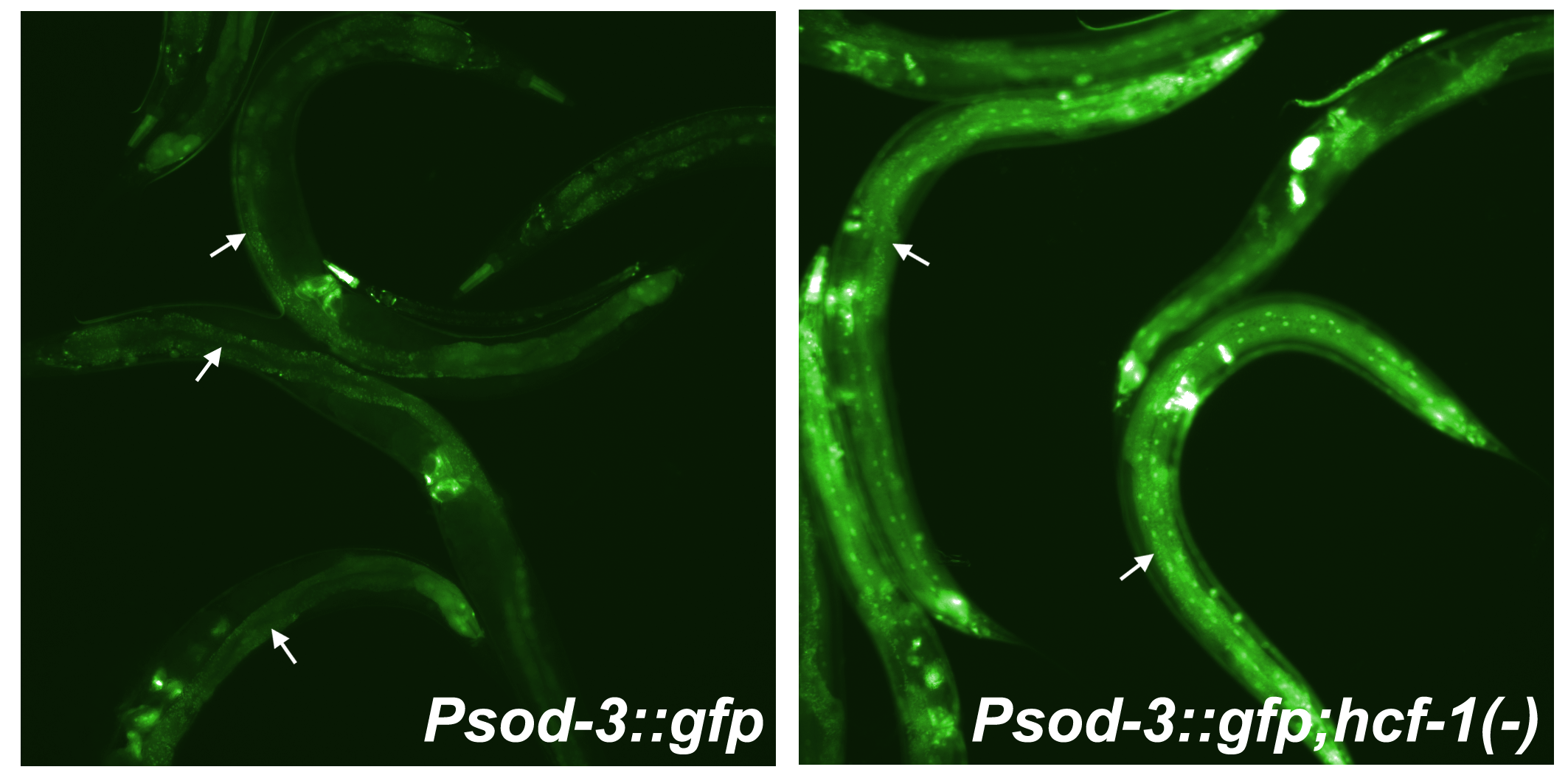

Supplement: Figure S5 — The GFP levels of Psod-3::gfp (muIs84[Psod-3::gfp]) in hcf-1(pk924) mutant was elevated (right panel) compared to that in wild-type background (left panel). Synchronized day 2 adults were shown in the photos. Arrowhead indicates the intestinal autofluorescence. (1.20 MB TIF) [file pbio.0060233.sg005.tif]

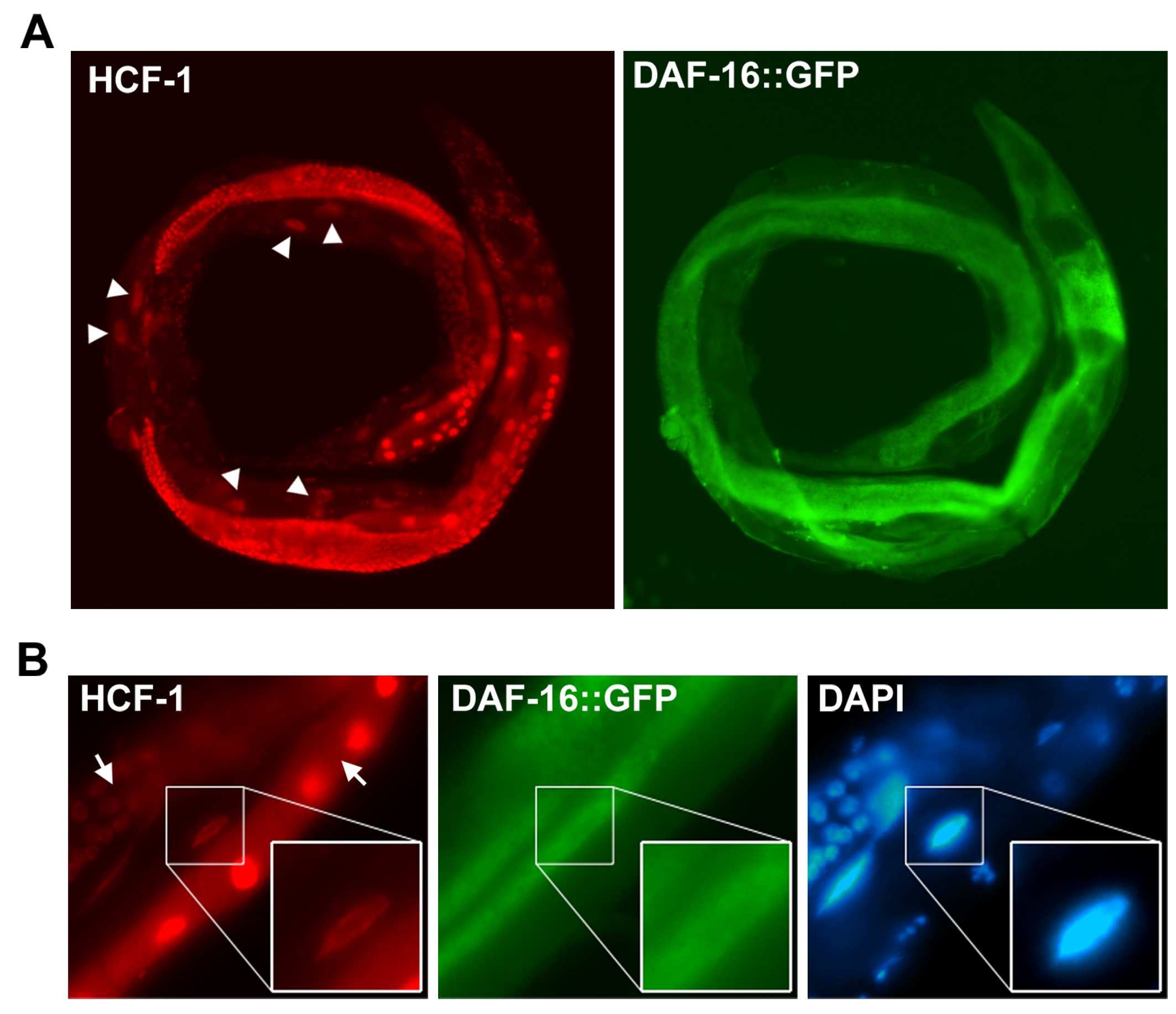

Supplement: Figure S6 — Transgenic worms over-expressing DAF-16::GFP (daf-16(mgDf47);xrIs87) were immunostained with anti-HCF-1. Photos were taken at 100× (A) or 400× (B) magnification and representative images are shown. Under normal culturing condition, DAF-16::GFP was diffusely localized in the cytoplasm and nucleus as previously reported [37,52]. HCF-1 co-localized with DAF-16::GFP in the nucleus of nongermline cells. DAPI staining was used to indicate the nucleus. Arrowheads indicate the nucleus of intestinal cells. Arrows indicate the gonad of the worms where DAF-16::GFP expression is absent as a result of transgene silencing [68]. (1.70 MB TIF) [file pbio.0060233.sg006.tif]
