# Supplementary material for: Caenorhabditis elegans HCF-1 Functions in Longevity Maintenance as a DAF-16 Regulator
Source: PLoS Biol. 2008 Sep 30;6(9):e233. doi: 10.1371/journal.pbio.0060233 (PMC2553839; doi:10.1371/journal.pbio.0060233)
Supplement: Table S1 — (53 KB DOC) [file pbio.0060233.st001.doc]

**Supplemental Table 1 Inactivation of *hcf-1* results in lifespan increase that is completely dependent on *daf-16*, but likely independent of the IIS pathway.**

| **Strain** | **Mean LS ± SEM (Days)** | **Total Number of Animals Died/Total** | **% of wt** | **p Value versus wt** | | **p Value versus *daf-16(mgDf47);hcf-1(ok559)*** | |
| --- | --- | --- | --- | --- | --- | --- | --- |
| wt | 13.1±0.5 | 34/34 |  | N.A. | | <0.0001 | |
| *daf-16(mgDf47)* | 10.8±0.3 | 38/38 | 82% | <0.0001 | | 0.8135 | |
| *hcf-1(ok559)* | 18.0±0.8 | 38/38 | 137% | <0.0001 | | <0.0001 | |
| *daf-16((mgDf4);hcf-1(ok559)* | 11.0±0.3 | 41/41 | 84% | <0.0001 | | N.A. | |
|  | | | | | | | |
| **Strain + RNAi** | **Mean LS ± SEM (Days)** | **Total Number of Animals Died/Total** | **% of wt+L4440** | **p Value versus wt + L4440** | **p Value versus *hcf-1(ok559)* + *daf-16*** | | |
| wt + L4440 | 13.6±0.3 | 48/48 |  | N.A. | <0.0001 | | |
| wt + *daf-16* | 9.9±0.2 | 28/28 | 73% | <0.0001 | 0.1278 | | |
| *hcf-1(ok559)* + L4440 | 16.3±0.6 | 27/27 | 120% | <0.0001 | <0.0001 | | |
| *hcf-1(ok559)* + *daf-16* | 10.3±0.2 | 36/36 | 76% | <0.0001 | N.A. | | |
|  | | | | | | | |
| **Strain** | **Mean LS ± SEM (Days)** | **Total Number of Animals Died/Total** | **% of wt** | **p Value versus wt** | | **p Value versus *daf-2(e1370);hcf-1(ok559)*** | |
| wt | 13.9±0.4 | 46/50 |  | N.A. | | <0.0001 | |
| *daf-2(e1370)* | 30.3±1.3 | 50/50 | 218% | <0.0001 | | 0.0112 | |
| *hcf-1(ok559)* | 18.6±0.6 | 50/50 | 134% | <0.0001 | | <0.0001 | |
| *daf-2(e1370);hcf-1(ok559)* | 35.9±1.0 | 51/51 | 257% | <0.0001 | | N.A. | |
|  | | | | | | | |
| **Strain + RNAi** | **Mean LS ± SEM (Days)** | **Total Number of Animals Died/Total** | **% of wt + L4440** | **p Value versus wt + L4440** | | | **p Value versus *hcf-1(ok559)* + *daf-2*** |
| wt + L4440 | 14.5±0.2 | 118/118 |  | N.A. | | | <0.0001 |
| wt + *daf-2* | 27.4±0.4 | 38/38 | 189% | <0.0001 | | | <0.0001 |
| *hcf-1(ok559)* + L4440 | 16.0±0.5 | 37/37 | 110% | <0.0001 | | | <0.0001 |
| *hcf-1(ok559)* + *daf-2* | 30.2±0.4 | 38/38 | 208% | <0.0001 | | | N.A. |
|  | | | | | | | |
| **Strain + RNAi** | **Mean LS ± SEM (Days)** | **Total Number of Animals Died/Total** | **% of wt + L4440** | **p Value versus wt + L4440** | | **p Value versus *hcf-1(ok559)* + *age-1*** | |
| wt + L4440 | 14.5±0.2 | 118/118 |  | N.A. | | <0.0001 | |
| wt + *age-1* | 21.1±0.3 | 77/78 | 146% | <0.0001 | | <0.0001 | |
| *hcf-1(ok559)* + L4440 | 16.0±0.5 | 37/37 | 110% | <0.0001 | | <0.0001 | |
| *hcf-1(ok559)* + *age-1* | 26.5±0.5 | 44/44 | 183% | <0.0001 | | N.A. | |

The lifespan experiments were repeated at least two independent times with similar results and the data for representative experiments are shown. The lifespan data were analyzed using the Log-rank test and p values for each individual experiment are shown.

N.A.: not applicable.
