# Supplementary material for: Caenorhabditis elegans HCF-1 Functions in Longevity Maintenance as a DAF-16 Regulator
Source: PLoS Biol. 2008 Sep 30;6(9):e233. doi: 10.1371/journal.pbio.0060233 (PMC2553839; doi:10.1371/journal.pbio.0060233)
Supplement: Table S2 — (33 MB DOC) [file pbio.0060233.st002.doc]

**Supplemental Table 2 Expression of a *hcf-1::gfp* transgene partially rescues the lifespan phenotype of *hcf-1(pk924)*.**

| **Strain + RNAi** | **Mean LS ± SEM (Days)** | **Total Number of Animals Died/Total** | **% of wt + L4440** | **p Value versus *hcf-1(pk924)* + L4440** | **p Value versus *hcf-1(pk924);hcf-1::gfp* +*hcf-1*** |
| --- | --- | --- | --- | --- | --- |
| wt + L4440 | 18.0±0.2 | 120/120 |  | <0.0001 | <0.0001 |
| *hcf-1(pk924)* + L4440 | 23.6±0.3 | 115/120 | 131% | N.A. | 0.0396 |
| *hcf-1(pk924)* + *hcf-1* | 24.2±0.3 | 115/120 | 134% | 0.3808 | 0.0020 |
| *hcf-1(pk924);hcf-1::gfp* + L4440 | 20.3±0.3 | 119/120 | 113% | <0.0001 | <0.0001 |
| *hcf-1(pk924);hcf-1::gfp* + *hcf-1* | 22.7±0.3 | 117/120 | 126% | 0.0396 | N.A. |

Expression of *hcf-1::gfp* in *hcf-1(pk924)* mutant resulted in partial suppression of the long lifespan phenotype. The lifespan suppression caused by *hcf-1::gfp* expression could be reverted by treating the *hcf-1(pk924);hcf-1::gfp* transgenic worms with *hcf-1* RNAi, whereas *hcf-1* RNAi had no effect on the long lifespan of *hcf-1(pk924)* mutant worms, supporting that the reduced lifespan observed in the *hcf-1(pk924);hcf-1::gfp* worms was due to expression of wild-type HCF-1 and that the *hcf-1::gfp* transgene is functional. *hcf-1(pk924);hcf-1::gfp* referred to *hcf-1(pk924);rwIs3[Phcf-1::hcf-1::gfp,unc-119]*.
